# Cyclodextrin Inclusion Complexes with Hydrocortisone-Type Corticosteroids

**DOI:** 10.3390/pharmaceutics16121544

**Published:** 2024-12-02

**Authors:** Aleksandra Kowalska, Łukasz Szeleszczuk

**Affiliations:** 1 Department of Organic and Physical Chemistry, Faculty of Pharmacy, Medical University of Warsaw, Banacha 1 Str., 02-093 Warsaw, Poland; aleksandra.kowalska@wum.edu.pl; 2 Doctoral School, Medical University of Warsaw, Żwirki i Wigury 81 Str., 02-093 Warsaw, Poland

**Keywords:** cyclodextrin, inclusion complex, corticosteroid, hydrocortisone, cortisol, CD, host–guest complexes

## Abstract

The hydrocortisone-type corticosteroid (HTC) group includes valuable active pharmaceutical ingredients (APIs) such as hydrocortisone, hydrocortisone acetate, cortisone acetate, tixocortol pivalate, prednisolone, methylprednisolone, and prednisone. Unfortunately, those APIs are characterized by low solubility, which hampers their application and reduces their therapeutic efficacy. The low polarity of HTC molecules allows them to form inclusion complexes with various cyclodextrins (CDs); however, as shown in this review, the type of applied CDs has a major impact on the final properties of the formed complex. HTC–CD complexes have been routinely used for over 40 years to achieve various aims. Most frequently, CDs have been utilized as HTC solubilizers and absorption enhancers in pharmaceutical formulations, as well as for separation and analysis by chromatographic and electrophoretic methods. This article reviews the studies describing the synthesis as well as the biological, physiochemical, and structural properties of the inclusion complexes formed between HTC and various cyclodextrins.

## 1. Introduction

Cyclodextrins (CDs) are cyclic oligosaccharides composed of α-1,4-linked glucopyranose units that have been extensively studied for their ability to form inclusion complexes with various active pharmaceutical ingredients (APIs), including corticosteroids [1]. Corticosteroids is a class of steroid hormones that includes both endogenous compounds produced in the adrenal cortex of vertebrates, as well as their synthetic analogues. Corticosteroids are used in a variety of conditions, ranging from hematological neoplasms to brain tumors or skin diseases. They are grouped into four classes, based on their chemical structure, which is known as the “Coopman classification”. Hydrocortisone (HC), also known as cortisol, is a naturally occurring glucocorticoid hormone that is widely used in the treatment of inflammatory conditions and allergic reactions [2]. It is also a key member of the Coopman’s Group A—hydrocortisone-type corticosteroids (HTCs). Unfortunately, most HTCs have a low aqueous solubility, which can limit their bioavailability and therapeutic efficacy [3].

**Figure 1:**
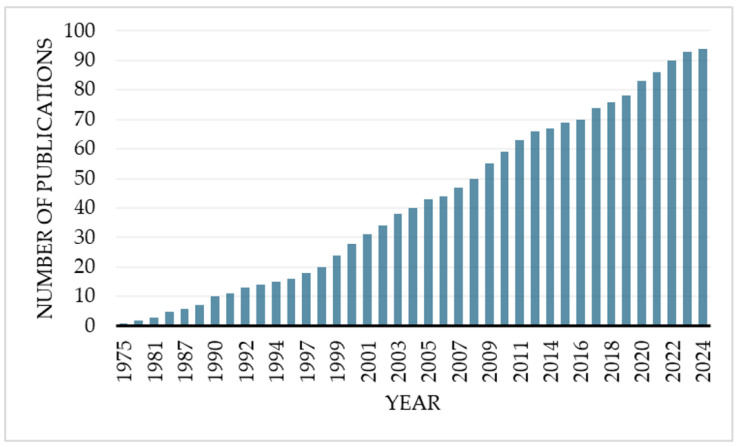
Number of published original works describing cyclodextrin (CD) inclusion complexes with hydrocortisone-type corticosteroids (HTCs). Each column shows the number of articles in the given year and all years before. For example, the column entitled “2011” depicts the number of articles published in the period 1975–2011, including 2011.

The formation of inclusion complexes between CDs and HTC has been investigated as a strategy to improve the solubility, stability, and bioavailability of those APIs [1]. CDs can encapsulate the hydrophobic steroid molecule within their hydrophobic cavity, while the hydrophilic exterior of the CD enhances the aqueous solubility of the complex [1]. Various types of CDs, most frequently β-CD and its derivatives, have been studied for their ability to form inclusion complexes with HTCs. Because CDs have become more and more popular in recent years, there is a large and diverse body of literature addressing their use in conjunction with HTCs (Figure 1). To the best of our knowledge, though, no reviews have been published specifically about the complexes of CDs and this group of APIs.

This review aims to provide a comprehensive overview of the current state of knowledge on CD/hydrocortisone-type corticosteroid inclusion complexes, focusing on the various types of CDs used, the physicochemical characterization of the complexes, and their applications.

To facilitate the reception of this review, rather than dividing the analysis of the articles into sections that explain different characteristics of the complexes, we chose to write distinct paragraphs that concentrate on the specific compounds from the HTC group. As a result, every paragraph in this review’s main body is structured similarly, providing comprehensive details on the procedures used to synthesize and analyze the CD-based complexes containing a specific HTC molecule.

## 2. Design and Methodology of the Study

Two independent examiners (A.K. and Ł.S.) were selected to choose the articles for the purpose of this review. The examiners performed a comprehensive literature search regarding the studies on the inclusion complexes formed between CDs and HTC in Scopus and PubChem databases. The following keywords and their synonyms were included among the search terms: “cyclodextrin” with combination of any of “HCT”, “hydrocortisone”, “cortisone”, “tixocortol”, “prednisolone”, “methylprednisolone”, and “prednisone”.

**Figure 2:**
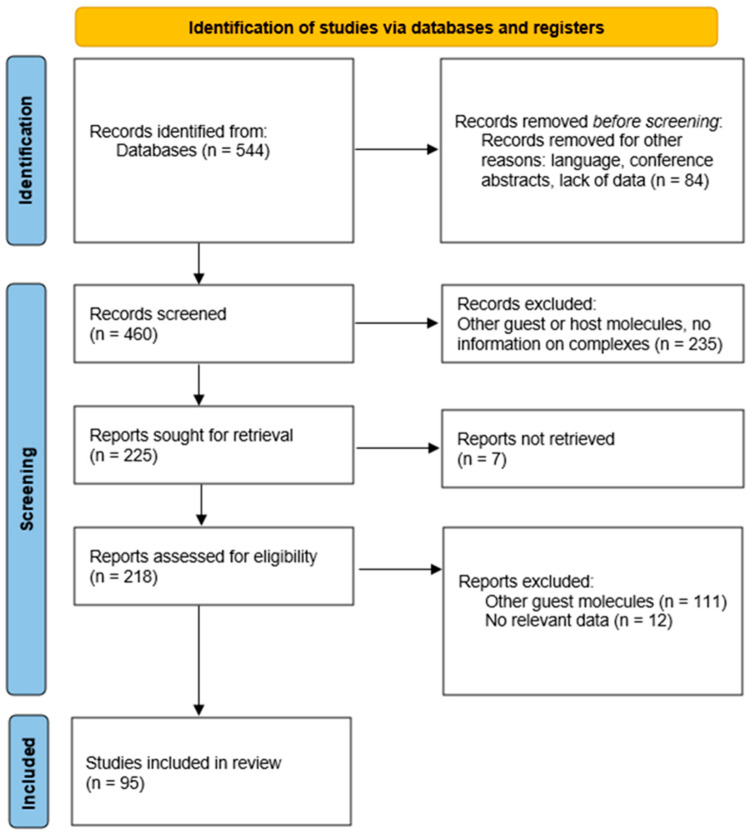
Flow diagram according to PRISMA statement.

Hence, the inclusion criteria for this review were the application of both native and substituted CDs as host molecules to form the inclusion complexes with HTCs. The exclusion criteria for this study were the use of either other complexing agents or APIs not from the HCT group. After finishing the inclusion and exclusion process, any disagreements were handled by the consensus between the reviewers. A thorough review has been conducted of the included publications to identify any further relevant research that may be included in the review. The flowchart based on the Preferred Reporting Items for Systematic Reviews (PRISMA) statement has been presented in Figure 2.

## 3. Corticosteroids

### 3.1. Chemical Structures of Hydrocortisone-Type Corticosteroids

**Figure 3:**
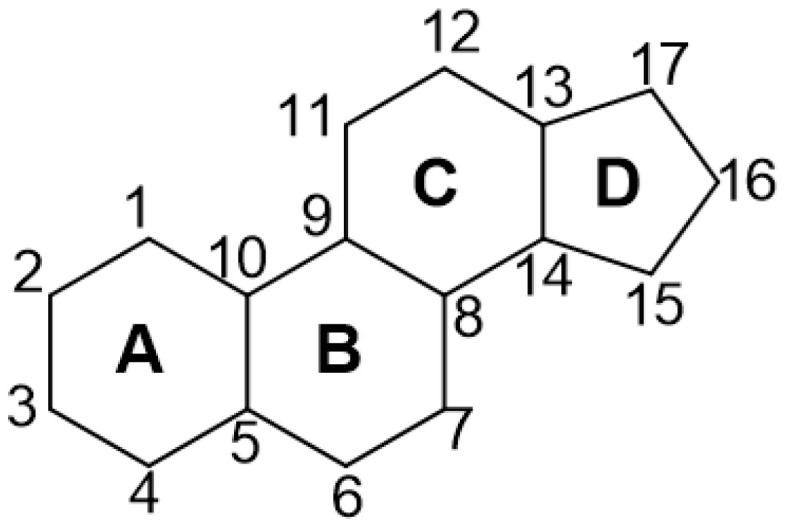
Basic steroid structure with IUPAC-approved ring lettering and carbon atom numbering.

The gonane (cyclopentaperhydrophenanthrene) unit is a structural feature shared by all steroids, including corticosteroids. The gonane unit is composed of four fused rings, namely, two cyclohexane rings, one cyclopentane ring, and one cyclohexadienone ring (Figure 3).

**Figure 4:**
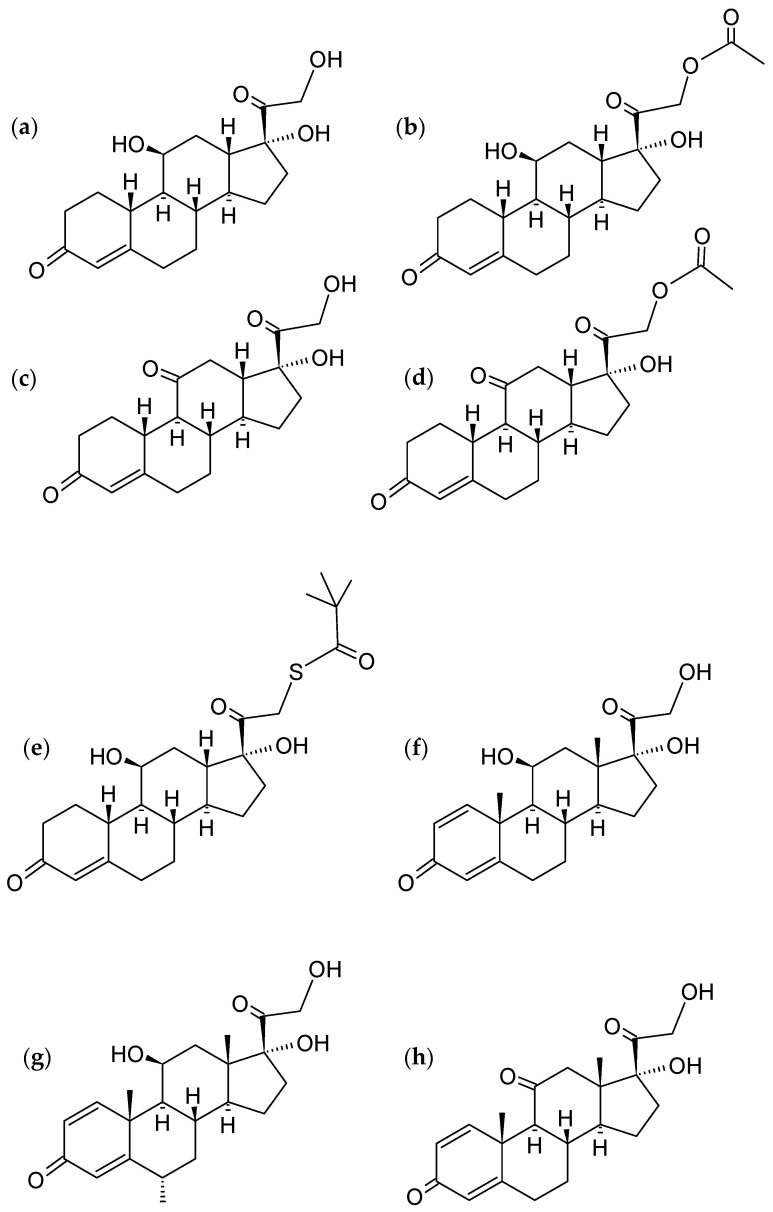
Chemical structures of hydrocortisone-type corticosteroids (HTCs). (**a**) Hydrocortisone, (**b**) hydrocortisone acetate, (**c**) cortisone, (**d**) cortisone acetate, (**e**) tixocortol pivalate, (**f**) prednisolone, (**g**) methylprednisolone, and (**h**) prednisone.

Based on their chemical structures and patch test results, corticosteroids are generally divided into four classes: hydrocortisone type (HTC, group A), acetonides with related substances (group B), betamethasone type (group C), and esters (group D). Regrettably, this categorization excludes systemic corticosteroids, but it does apply to topical steroids and offers a systematic summary of cross-reactivity; therefore, physicians can use it as a reference when selecting a substitute corticosteroid [4]. The HTC group partially overlaps with the Anatomical Therapeutic Chemical Classification System (ATC) group D07AA, which includes hydrocortisone, prednisolone, and methylprednisolone [5]. The chemical structures of HTC are presented in Figure 4 [4]. Among the compounds in the HTC group, there are no available data on the formation of cyclodextrin complexes solely with tixocortol pivalate.

### 3.2. Biological Functions of Corticosteroids

Corticosteroids, a class of steroid hormones produced by the adrenal cortex, possess various biological functions. The adrenal cortex is composed of up of three cellular zones, each of which produces a specific type of steroidal hormones. Their synthesis starts with cholesterol and ends with the synthesis of androgens, glucocorticoids, and mineralocorticoids. The primary glucocorticosteroid, cortisol, is responsible for numerous physiological processes, including gluconeogenesis, the process that corresponds to the term “glucocorticosteroid” [5], but also stress response, carbohydrate metabolism, protein catabolism, inflammation, and immune modulation [6].

Due to their lipophilic nature, glucocorticoids must be quickly produced (via a variety of enzymatic processes) in response to acetylcholine stimulation rather than being presynthesized and stored in the adrenal glands. Although adrenal glucocorticoid production maintains the level of glucocorticoids in the systemic circulation, the tissue or cellular regulation of glucocorticoid availability is also present. The majority of circulating glucocorticoids in humans are kept in an inactive form by binding 80% to 90% of them to corticosteroid-binding globulin (CBG) and 5% to 15% of them to albumin. Merely 5% of glucocorticoids in the system are free and bioactive [7].

### 3.3. Pharmacological Importance of Glucocorticosteroids

Several glucocorticoid administration methods are employed in clinical settings, contingent upon the nature of the illness, the implicated organ, and the degree of participation. Patients with severe disorders involving major organs typically require high dosages of daily glucocorticoids; patients with less aggressive diseases may use alternate-day regimens. Patients with immune-mediated disorders that advance quickly often begin therapy with intravenous glucocorticoids, often known as pulse therapy [8].

Glucocorticosteroids, due to their robust anti-inflammatory and immunosuppressive actions, are essential in treating a variety of medical conditions such as the following:

- Autoimmune Diseases: Conditions like rheumatoid arthritis, lupus, and multiple sclerosis benefit from glucocorticosteroids, which reduce immune response overactivity, alleviating symptoms and inflammation. Drugs such as prednisone and prednisolone are commonly used for these conditions.
- Respiratory Disorders: In asthma and chronic obstructive pulmonary disease (COPD), glucocorticosteroids (e.g., methylprednisolone and prednisone) help to reduce airway inflammation and improve breathing, especially during flare-ups.
- Allergic Reactions and Dermatological Conditions: Corticosteroids treat severe allergic reactions and inflammatory skin conditions, such as eczema and psoriasis. Hydrocortisone and tixocortol are particularly effective in topical and systemic forms.
- Endocrine Disorders: In adrenal insufficiency, hydrocortisone is used to replace deficient cortisol, a critical hormone for maintaining blood pressure, metabolism, and stress response.
- Organ Transplantation: To prevent rejection, glucocorticosteroids (like prednisone) are integral to immunosuppressive regimens, decreasing immune-mediated damage to transplanted organs.
- Cancer Therapy Support: Glucocorticosteroids help to manage side effects of chemotherapy, such as nausea and inflammation, and are used in certain leukemias and lymphomas to reduce white blood cell production and manage inflammation.

Each glucocorticosteroid is selected based on pharmacokinetic properties like bioavailability and the duration of action, ensuring that treatment aligns with the specific needs of each condition [9].

## 4. Cyclodextrins and Their Inclusion Complexes with Steroids

The core of the structure of cyclodextrins (CDs), a class of molecules composed of α-D-glucopyranose units, linked by the α-1,4 glycosidic bonds, is a hydrophobic cavity that can enclose other compounds. The remarkable encapsulating properties of CDs lead to the development of a “host–guest” relationship that modifies or improves the physical, chemical, or biological characteristics of the guest molecule. Those complexes, while usually equimolecular, can sometimes exist at different guest–host molar ratios, i.e., 1:2, 1:3, etc. To avoid the misunderstanding, in this review, the molar ratios are always presented as guest to host (API:CD) (Table 1). Although the most commonly utilized cyclodextrins are naturally occurring α, β, and γ, which consist of six, seven, and eight glucose subunits, respectively, large-ring cyclodextrins (LR-CDs), with nine to more than several hundred units, have also been studied and used [10]. Furthermore, because of their unique properties, CD derivatives have been also widely used in a wide range of industries, including the food, cosmetic, and biomedical sectors [11]. Undoubtedly, CDs are valuable excipients in medications that are administered orally, topically, nasally, or rectally [12]. Additionally, for the oral formulations, the complexation of API with CDs can alter the enzymatic breakdown and subsequent pathways of drug absorption due to the changes in the concentrations at the various stages of gastrointestinal track, which may influence the pharmacokinetic properties.

The extremely low water solubility of steroidal APIs poses a challenge to their administration. Thus, complexation with various CDs is one of the methods to increase those hydrophobic APIs’ bioavailability [1]. Because steroids are similar in their sizes to CD’s cavities and are hydrophobic, they are excellent guest molecules for complexation by cyclodextrins.

Apart from native CDs, multiple CD derivatives, such as hydroxypropyl HP-β-CD and sulfobutyl ether (SBE)-β-CD, have been developed and investigated for their enhanced solubility, ability to form complexes, and toxicological characteristics, demonstrating their significant utility. Additional alterations involve the replacement of one or more primary hydroxyl group with glucosyl or maltosyl groups through an α-(1→6) glycosidic linkage at the narrow rim of the CD [40]. Specifically, in the sulfoalkyl ether derivatives (SAE-CD), the addition of an anionic substituent greatly enhances the ability of the parent CD to dissolve in water and reduces its potential for causing kidney damage, hence improving its safety profile [51].

Phase solubility studies assess the influence of CD concentration on drug solubility behavior. The API–CD complexes exhibiting A-type curves demonstrate partial water solubility, typically linked to water-soluble CD derivatives. The A_L_-subtype curve illustrates a linear connection between drug and CD concentrations, applicable to complexes with a 1:1 stoichiometry. The creation of higher-order complexes may disrupt linearity. The positive deviation—A_P_-subtype curve—is characteristic of complexes of API:CD 1:2, 1:3, etc., where an increase in the amount of CD results in a better solubility improvement in comparison with the 1:1 complexes, whereas the negative deviation—A_N_-subtype curve—occurs for API:CD 2:1, 3:1, etc., for which a further increase in the used CD is hindered and approaches a plateau. The B-type curve signifies API-CD complexes exhibiting reduced water solubility. The B_S_-subtype curve is characteristic of complexes with restricted water solubility, where an initial rise in solubility is succeeded by complex precipitation, leading to a plateau in the curve and a subsequent decline in solubility. Insoluble compounds exhibit B_I_-subtype curves, which mirror the trajectory of B_S_-subtype curves, albeit lacking the early solubility increase.

## 5. Hydrocortisone-Type Corticosteroids That Form Host–Guest Complexes with Cyclodextrin

### 5.1. Hydrocortisone

#### 5.1.1. Complex Preparation, Structural Studies, and Solubility

The chronologically first article on the interactions between the HTC and CD was published in 1982. In this work the formation of an inclusion complex between β-cyclodextrin and hydrocortisone (HC) has been investigated using proton nuclear magnetic resonance (^1^H-NMR) and phase solubility studies. The extent of the chemical changes of the interior and exterior protons of β-cyclodextrin in the presence of HC indicated that HC is encapsulated within the β-cyclodextrin cavity and likely interacts with hydrogen atoms at the periphery of the its toroidal structure. While the stoichiometry of the β-CD/HD complex could not have been definitively established from ^1^H-NMR analysis, the pronounced chemical shift changes of hydrocortisone’ H-3 and H-5 indicated that a segment of one HC molecule is tightly accommodated within the cavity in close proximity to these protons. This observation was corroborated by a basic molecular modeling of the inclusion complex, indicating that multiple molecules of HC per one molecule of β-CD (each partially situated within the cavity) would yield an inadequate fit for either guest molecule, resulting in a loosely bound complex. A phase solubility study revealed that the inclusion compound did not exhibit a simple 1:1 stoichiometry; rather, the characteristics of the complex appeared to be contingent upon the overall concentration of hydrocortisone in relation to that of β-CD. The overall stoichiometry of the inclusion compound was complex and varied, seemingly contingent upon the relative quantities of hydrocortisone and β-CD in the system [52].

To further investigate the stoichiometry of the β-CD/HC complex, another research study was conducted using 10 mL liquid scintillation vials set in a water bath. The water bath was thermostatically controlled at a temperature of 30 ± 0.5 °C. Hydrocortisone with a precisely determined quantity of 0.05 mmol was introduced into a vial. Subsequently, a vial was supplemented with 5 mL of an aqueous solution having varying doses of β-CD, spanning from 0.01 to 25 mM. Afterwards, the mixtures were stirred vigorously using a small magnetic stirrer for a period of 16 h. The solid in the equilibrated solution was isolated through filtration using a 0.4 μm polycarbonate nucleopore membrane. The remaining 2 mL of the filtered liquid was gathered, mixed with water, and analyzed using UV–Vis spectroscopy at 243 nm. The leftover solid was subjected to vacuum dehydration at a temperature of 65 °C using phosphorus pentoxide [14]. The stoichiometric ratio of the complexes was determined using three models: (a) phase solubility diagram, (b) fast atom bombardment mass spectral analysis of pure complexes, and (c) chemical examination of the pure solid complexes [14]. The equilibrium solubility investigations have revealed B-type behavior in the β-CD/HC systems, as indicated by the solubility phase diagrams. The solubility phase diagram’s early segment exhibits a notable rise in the overall concentration of steroids due to complexation. The early rise in steroid solubility is significantly higher compared to the solubility found later in the solubility phase diagram at high β-CD concentrations. The solubility becomes similar to that of the pure 1:2 steroid complex when the β-CD concentration exceeds 20 mM. The results suggest that the complexes in the solid state have a stoichiometric ratio of 1:2. The FAB mass spectra reveal the presence of both 1:1 and 1:2 complex molecular ions among the mass fragments collected [14].

The data suggest that the HC molecule initially forms an inclusion complex with one molecule of β-CD, which appears to be a more thermodynamically preferred process compared to the binding of the 1:1 complex with the second molecule of β-CD, as indicated by the magnitudes of K_1_ and K_2_. HC possesses a hydroxyl group at the 11th position, which can create an intramolecular hydrogen bond with the ketone group of the side chain at the 17th position. The development of the 1:1 complex disrupts the hydrogen bond and releases the secondary hydroxyl group at the 11th position. This leads to an elevation in the chemical polarity and a reduction in the capacity of the 1:1 complex to interact with another β-CD molecule. This discovery implies that the 1:1 combination first formed between the five-member cyclopentane ring of the steroid and a β-CD molecule. Next, the second step entails incorporating the free A ring into the second β-CD cavity to eventually create the 1:2 complex [14].

Despite its limited aqueous solubility and poor palatability, HC is often utilized off-label per os in pediatric therapy. Consequently, a reconstitutable hydrocortisone solution with a disguised taste, capable of facile extemporaneous production, was formulated utilizing cyclodextrins. The excipients for the reconstitutable dry powder combination were chosen based on their solubility in water, compatibility, apparent non-toxicity, and stability. The formulation was produced in a citric acid buffer at pH 4.2, ensuring optimal pH for maximum HC stability. This study selected HP-β-CD due to its superior solubility relative to the parent β-CD [53]. 

Pre-formulation tests were conducted to determine the optimal HC:HP-β-CD molar ratio for rapid reconstitution. The formulation containing the minimal quantity of cyclodextrin, specifically HC:HP-β-CD at a molar ratio of 1:2, was inadequate for achieving complete solubilization, resulting in a suspension. Crystal formation was noted despite hand shaking the HC:HP-β-CD (1:4) inclusion complex for almost 5 min. Alternative formulations incorporating HC:HP-β-CD at molar ratios of 1:7, 1:9, and 1:10 resulted in the development of a viscous paste, perhaps attributable to the substantial quantity of HP-β-CD. The paste may be predominantly solubilized by manual shaking for over 15 min, which would be difficult in a clinical environment. The elevation of HP-β-CD concentration did not enhance the reconstitution. The HP-β-CD (1:6) inclusion complex facilitated the complete and quick solubilization of HC upon reconstitution, hence mitigating the risk of dosage errors associated with suspensions. HP-B-CD established a complex with HC at a molar ratio of 1:6, enhancing the drug’s apparent solubility by about 20-fold. HP-β-CD was predominantly utilized to improve the inadequate solubility of HC. Given that cyclodextrins can also mitigate bitterness, it was anticipated that HP-β-CD might demonstrate a synergistic effect with the sweetener, which was eventually confirmed [53].

The molecular mechanisms responsible for the drug-solubilizing capabilities of γ-CD were investigated utilizing HC as a model compound. The B_S_-type phase-solubility diagram of HC with γ-CD was meticulously characterized by quantifying the concentrations of HC and γ-CD in both the solution and solid phases. The interaction between the drug and solubilizer was examined using isothermal titration calorimetry, yielding an accurate 1:1 binding constant (K_11_ = 4.01 mM^−1^ at 20 °C). The formation of water-soluble 1:1 complexes accounts for the initial rise in HC solubility, whereas the precipitation of 2:3 HC/γ-CD entities leads to the plateau and subsequent significant decline in solubility after the complete utilization of solid hydrocortisone. The comprehensive phase solubility diagram is accurately represented by a model utilizing the 1:1 binding constant and the solubility constant of the precipitating 3:2 species (K_23_^S^ = 5.51 mM^5^). In such systems, a slight excess of γ-CD over the optimal concentration may lead to a notable reduction in drug solubility [27].

The solubilization boost of HC by β-CD is improved by the incorporation of short-chain anionic and cationic species in the aqueous complexation medium. The maximal solubility of HC in pure aqueous β-CD solutions or suspensions is 2.2 mg/mL. The incorporation of 1% (*w*/*v*) sodium acetate into the complexation medium elevates this value to 7.1 mg/mL, representing an increase of approximately 220%. The subsequent incorporation of 0.25% (*w*/*v*) hydroxypropyl methylcellulose into the solution enhanced the solubility of HC to over 9 mg/mL. Analogous findings were seen upon the addition of sodium salicylate or benzalkonium chloride to the complexation solution. The authors have suggested that the development of simple API/CD inclusion complexes does not adequately account for the solubilizing activities of CDs. The creation of non-inclusion complexes, including water-soluble microaggregates of APIs and API/CD complexes, can enhance overall solubility. In aqueous environments, water-soluble polymers like HPMC, together with anionic and cationic molecules, seem to augment the role of non-inclusion complexation in total drug solubility while also solubilizing and stabilizing API/CD complexes [30]. To verify this hypothesis, three distinct methodologies were employed to examine the aggregation of API/CD complexes: drug permeation through semi-permeable membranes, the assessment of variations in the activity coefficients of API/CD complex solutions, and transmission electron microscopy (TEM). The examined aqueous solutions comprised γ-CD or HP-γ-CD, or their combinations, together with HC. The permeation investigations revealed that API/CD complex monomers (i.e., unaggregated complexes) predominated at CD concentrations below 5% (*w*/*v*). The production of aggregates progressively escalated with rising CD concentration until the whole rise in dissolved API/CD complexes was attributed to the creation of cyclodextrin aggregates. This occurred despite the fact that the measured phase-solubility diagrams were linear, namely, of A_L_-type. The activity coefficients exhibited a positive divergence from the ideal condition. This positive deviation results from the concurrence of various processes, namely, hydration, aggregation, and complex formation. The noted deviations from ideality suggested the formation of complex aggregates in the aqueous complexation medium. TEM images revealed the production of aggregates in both pure water CD solutions and in CD solutions saturated with HC. The dimensions of HC/γ-CD and HC/HP-γ-CD complex aggregates are around 10–50 nm and 10–80 nm, respectively. The overall diameter of the HC/(γ-CD/HP-γ-CD) complex mixtures much exceeds that of γ-CD and HP-γ-CD, reaching several hundred nanometers, and seems to result from the aggregation of smaller complex aggregates [31].

A rapid and straightforward high-performance liquid chromatographic (HPLC) approach utilizing a charged aerosol detector (CAD) was established for the quantification of γ-CD in aqueous solutions. The chromatographic system comprised a C18 column as the stationary phase and an aqueous mobile phase containing 7% (*v*/*v*) methanol. The calibration curve was established for γ-CD concentrations ranging from 0.005% to 1% (*w*/*v*). The detection and quantitation limits of γ-CD were 0.0001% and 0.0002% (*w*/*v*), respectively. The formation of CD aggregates in aqueous solution and their critical aggregation concentration (CAC) were assessed using both conventional dynamic light scattering (DLS) and permeation methods with HPLC-CAD for quantitative analysis of CD. The CAC of CD was established at 0.95% (*w*/*v*), and the quantity of CD self-aggregates escalated with higher CD concentrations. The established HPLC-CAD method was employed to ascertain the γ-CD phase-solubility profile in an aqueous hydrocortisone HC/γ-CD complexation medium. The peak concentrations of dissolved γ-CD and HC were established at 1.47% and 0.31% (*w*/*v*), respectively. The membrane permeation technique proved to be a dependable approach for the assessment of metastable γ-CD aggregates. The HPLC-CAD approach was effectively utilized for the quantitative analysis of γ-CD in aqueous solutions during permeation and phase-solubility investigations [28].

**Figure 5:**
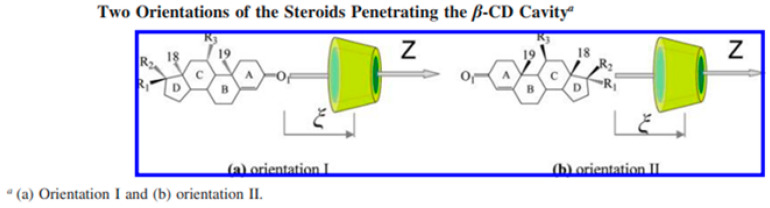
The orientations of the steroids penetrating the β-CD cavity. Reprinted with permission from [32]. Copyright 2009 American Chemical Society.

The incorporation of HC into the cavity of β-CD, following two potential orientations, was examined by molecular dynamics simulations and free-energy assessments. The free-energy profiles characterizing the inclusion process were established via an adaptive biasing force [32]. Two potential orientations of the steroid in relation to the wider rim of β-CD were examined: orientation I, involving the A ring, and orientation II, involving the D ring, as illustrated in Figure 5 [32].

**Figure 6:**
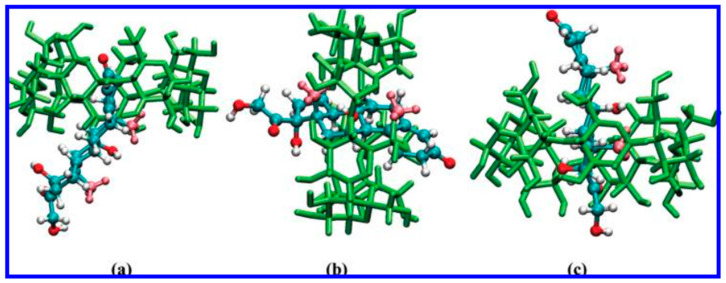
Snapshots of the inclusion complexes of Hyc with β-CD at the inflection points along the free-energy curve in orientation I. (**a**) Near the first minimum, ca. ξ = 1.9 Å; (**b**) near the maximum, ca. ξ = 6.0 Å; (**c**) near the second minimum, ca. ξ = 7.7 Å. Reprinted with permission from [32]. Copyright 2009 American Chemical Society.

**Figure 7:**
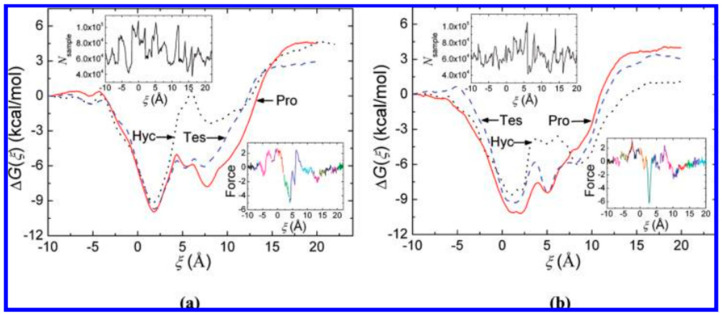
Free-energy profiles for the inclusion of steroids into β-CD cavity along the z axis in (**a**) orientation I and (**b**) orientation II, Hyc (dot line), Pro (solid line), Tes (short dash line). (Inset) Number of samples and continuity of force. Reprinted with permission from [32]. Copyright 2009 American Chemical Society.

**Figure 8:**
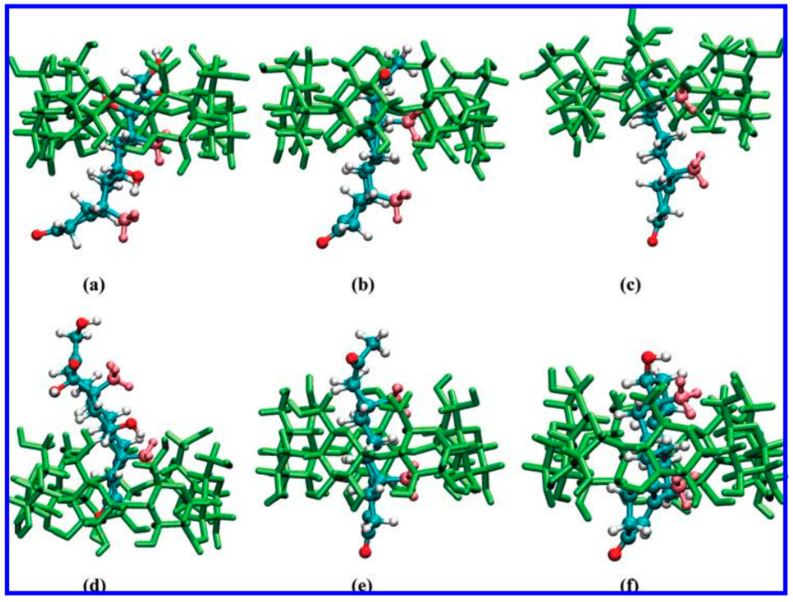
Snapshots of the inclusion complexes formed by the steroids and β-CD at the inflection points of the free-energy profile delineating association in orientation II. (**a**–**c**) Near the first minimum: (**a**) Hyc-β-CD, (**b**) Pro-β-CD, and (**c**) Tes-β-CD. (**d**–**f**) Near the second glaring minimum: (**d**) Hyc-β-CD, ca. ξ = 8.5 Å, (**e**) Pro-β-CD, ca. ξ =5.0 Å, and (**f**) Tes-β-CD, ca. ξ = 5.0 Å. Reprinted with permission from [32]. Copyright 2009 American Chemical Society.

**Figure 9:**
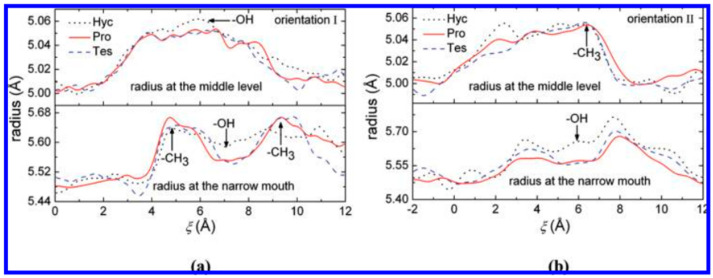
Fluctuation of the radius of the internal cavity as a function of the model reaction coordinate in (**a**) orientation I and (**b**) orientation II. (above) Radius in the middle of the cavity, determined by the seven glycosidic oxygen atoms. (below) Radius at the first rim of β-CD, determined by the seven ethyl carbon atoms at the primary side. Reprinted with permission from [32]. Copyright 2009 American Chemical Society.

In orientation I, where the A ring of the steroid enters the cavity of β-CD, the free energy decreases significantly to the first local minima at approximately 1.9 Å. The second prominent free-energy minimum associated with orientation I occurs at approximately 7.7 Å, corresponding to a profound inclusion structure, where the C19 methyl extends from the cavity but the C18 methyl does not reach the central plane. Figure 6 illustrates a representative structure. As HC distances itself from β-CD, the free energy gradually attains a plateau. Figure 7b illustrates the structure of the complexes at the local minima in orientation II. The distinct second minimum of HC is noted around 8.5 Å. In the structure depicted in Figure 8d, the C19 methyl group is positioned on the primary side, while the A ring is situated within the cavity, establishing advantageous van der Waals interactions. Furthermore, for HC, the free-energy barrier at 6.0 Å is lower than that in orientation I. This outcome arises from the observation that when the R3 hydroxyl group traverses the initial rim of the cavity, the C19 methyl group has yet to attain the core of β-CD; therefore, the associated steric hindrances are not intensified (refer to Figure 9b) [32].

The authors have concluded that orientation I seems to be preferred for the incorporation of HC into β-CD [32].

Investigations into the permeation of complexes across membranes and the relationships of phase solubility were conducted for saturated hydrocortisone solutions utilizing the parent cyclodextrins, specifically α-CD, β-CD, γ-CD, or various water-soluble derivatives, including 2-HP-β-CD, 2-HP-γ-CD, and SBE-β-CD. The data demonstrate that β-CD and γ-CD create micro-aggregates with hydrocortisone, leading to non-linear phase-solubility interactions. According to the contract, additional investigations of CDs or CD derivatives were conducted to create nanoaggregates with hydrocortisone, leading to linear solubilization correlations. The CDs exhibiting the least propensity to form complex aggregates were α-CD and SBE-β-CD, which was attributed to their poor complexation efficacy and repulsive forces, respectively. Complex aggregates with these CDs are also diminutive, with a maximum size ranging from 50 to 100 kDa. The HP-β-CD and HP-γ-CD complex aggregates had a maximum size above 100 kDa, and the proportion of the drug involved in complex aggregation with these species is greater than that of the other materials evaluated. In the 90 mM HP-γ-CD solution, statistics indicate that 87% of the hydrocortisone is associated with aggregates. TEM verified these elevated concentrations, revealing that the majority of particles were within the 3–5 nm range, with infrequent occurrences of particles measuring 10 and 20 nm. The apparent stability constant (K_1:1_) and the complexation efficiency (CE) were calculated (Table 2). For the three native cyclodextrins, both K_1:1_ and CE augmented with increasing cavity size; however, for the cyclodextrin derivatives, the substitution resulted in either elevated or diminished values. The researchers hypothesize that an increase in the cavity size of the CD correlates with a deeper penetration of the hydrocortisone molecule and an elevated CE [33].

In another work, the impact of temperature on the aggregation of HC/HP-β-CD complexes was examined, along with the effects of including ethanol or water-soluble polymers into the aqueous systems. Additionally, the impact of stirring on aggregation was evaluated. Size exclusion permeability analyses were performed to assess complex aggregation tendencies. The findings demonstrate that self-assembled complex aggregates are metastable and significantly decrease in size with rising temperature and the introduction of ethanol. Water-soluble polymers also diminish the dimensions of the complicated aggregates. Hexadimethrine bromide exerted the most significant effect, as its incorporation either eradicated aggregates from the systems or diminished their size below the molecular weight threshold of the sizing membrane (8 kDa). Comparable observations occur when aqueous solutions of HC and HP-β-CD are equilibrated through stirring. Analogous trends to those associated with ethanol addition can be noted by elevating the system temperature or by agitating the donor phase. All of these indicate that the forces involved in complex aggregation are rather feeble and can be readily disrupted. Nonetheless, the mechanical foundation of the intricate aggregation phenomena remains inadequately comprehended. This work suggests that hydrogen bonding may play a significant role in aggregate kinetics and thermodynamics. Nonetheless, this does not preclude other forms of weak non-covalent interactions, including van der Waals forces, electrostatic contacts, dipole interactions, charge transfer, the release of conformational strain, and hydrophobic interactions [54].

Another research aimed to examine the impact of prevalent pharmaceutical excipients and competing APIs on the solubilization of poorly water-soluble medicines and their bioavailability. An aqueous eye drop solution was utilized as a sample formulation containing dexamethasone as the model medication. The evaluated competitor API was HC, which possesses a comparable steroidal structure to dexamethasone. The sample CDs included γ-CD, which exhibits poor solubility in water, and its water-soluble derivative, HP-γ-CD. This was motivated by the results of prior research, which demonstrated that combinations of γ-CD and HP-γ-CD can serve as more effective solubilizers than the separate CDs. Consequently, combinations of γ-CD and HP-γ-CD were also incorporated into this investigation. The affinities of the two APIs to γ-CD and HP-γ-CD are comparable in magnitude, as indicated by the CE values in Table 3; however, HC generally exhibits a higher affinity for the CDs than dexamethasone. The CE is evidently diminished when both APIs coexist in the same complexation medium, leading to a reduction in the solubilization of dexamethasone and HC [34].

One of the studies focused on the complexation capability of new sulfobutyl ether-alkyl ether (SBE-AE-CD)-mixed β- and γ-CD derivatives with a series of structurally related steroids (6α-methylprednisolone, prednisolone, triamcinolone, D(-) norgestrel, and hydrocortisone). The impact of the total degree of substitution (TDS) and the length of the alkyl side chain on the binding capability of these newly modified CDs was assessed, as well as their potential to cause red blood cell hemolysis. The hemolysis percentage was evaluated by analyzing citrated rabbit blood and citrated human blood using UV analysis [35].

#### 5.1.2. Extraction and Analysis

Despite the limitations on the use of certain steroid hormones owing to their endocrine-disrupting properties, residues of these contaminants persist in various water matrices. Consequently, analytical approaches capable of identifying these chemicals within complicated matrices are urgently required. One study devised a straightforward and efficient magnetic solid-phase microextraction approach that demonstrated analytical performance superior to previous solid-phase extraction methods [55].

A β-CD-functionalized magnetic-activated carbon adsorbent was synthesized and characterized through multiple analytical techniques, including X-ray diffraction (XRD), scanning electron microscopy–electron diffraction spectroscopy (SEM-EDS), and TEM. This adsorbent was employed in the formulation of a magnetic solid-phase microextraction (MSPE) method for the preconcentration of estrone, β-estradiol, hydrocortisone, and progesterone in wastewater and river-water samples. This method was refined by central composite design to identify the experimental parameters influencing the extraction operation. The measurement of hormones was accomplished utilizing HPLC with a diode array detector (HPLC-DAD). Under optimal conditions, the linearity spanned from 0.04 to 300 µg L^−1^, with a correlation coefficient ranging from 0.9969 to 0.9991. The detection and quantification limits were from 0.01 to 0.03 µg L^−1^ and 0.033 to 0.1 µg L^−1^, respectively, with intraday and interday precisions of 1.1 to 3.4 and 3.2 to 4 [55].

The equilibrium data were optimally represented using the Langmuir isotherm model, yielding high adsorption capacities (217–294 mg g^−1^). The established approach exhibited considerable efficacy for application in wastewater samples with few interferences, and the adsorbent was reusable up to eight times [55].

#### 5.1.3. Diffusion and Permeability Studies

Cyclodextrins are acknowledged as effective carriers that solubilize hydrophobic APIs molecules and transport them to the biological membrane’s surface, such as the skin, mucosa, or cornea, where they can integrate into those membrane [56]. The researchers studied the mechanism underlying the observed improvement of CD penetration using hairless mouse skin and a semi-permeable membrane as a model (Equation (1)):

(1) $$J_{D}==\frac{(\frac{{P^{\prime }}_{M}}{K_{d}}){\left[D\cdot CD\right]}_{d}}{M_{1/2}+{\left[CD\right]}_{d}}$$

where J_D_ is the drug flux, M_1/2_ is the cyclodextrin concentration in saturated drug solutions where the flux is half of the maximum flux, [D∙CD]_d_ and [CD]_d_ are the concentrations in the donor phase of the drug–cyclodextrin complex (D∙CD) and cyclodextrin (CD), K_d_ is stability constant, and P′_M_ is the membrane permeability constant.

**Figure 10:**
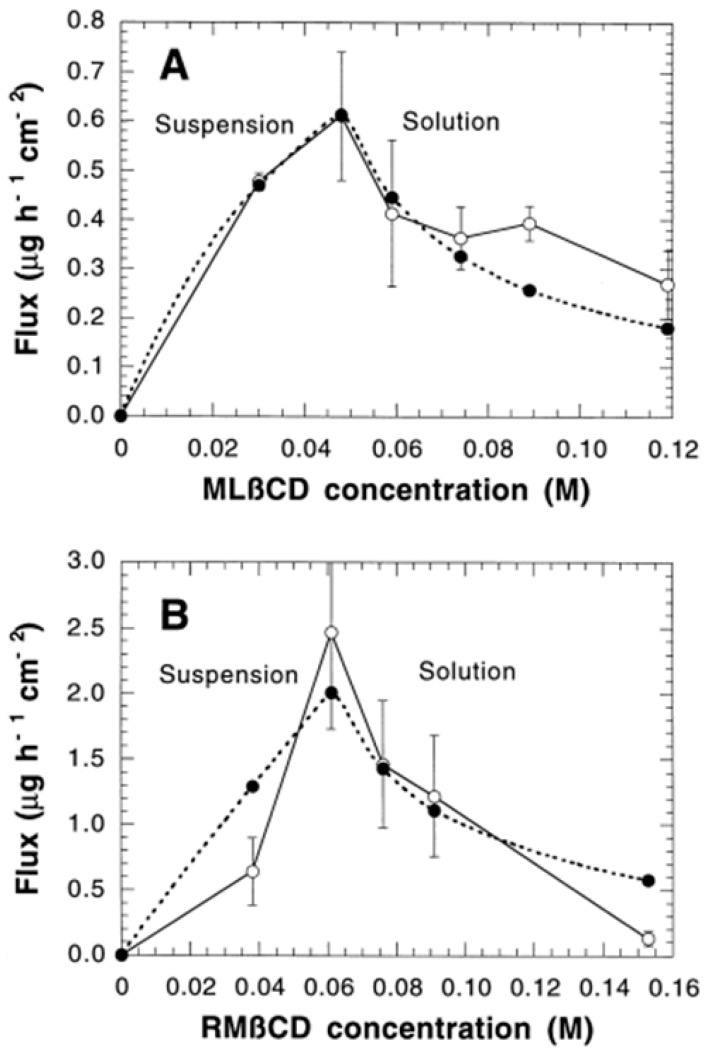
The effect of ML-β-CD (**A**) and RM-β-CD (**B**) concentration on the flux of hydrocortisone from aqueous solutions through hairless mouse skin. The solid line (◦) represents the experimental data, and the dash line (•) shows the results of fitting to Equation (1). Reprinted with permission from [57].

Figure 10 illustrates the flow of HC at varying concentrations of RM-β-CD (randomly methylated beta cyclodextrin) and ML-β-CD (maltosyl beta cyclodextrin). In both instances, the drug flow significantly escalated with increasing CD concentration when the API was in suspension but diminished when CD was in surplus. This general behavior aligned with Equation (1); however, a generally strong correlation with the experimental data was observed with the ML-β-CD. A sharper-than-anticipated increase to the maximum flow was reported in the case of RM-β-CD. The CM-β-CD and HP-β-CD M_1/2_ values ranged from 0.015 to 0.096 M for pure cyclodextrin solutions, while the P_M/K_ values ranged from 0.54 to 3.9 μM h^−1^ cm^−^². The pattern for these CDs resembled that observed with the RM-β-CD. The synthesis of CD drug complexes via thermal treatment in the presence of water-soluble polymers frequently results in the enhanced solubility of both the drug and the CD. The bioavailability of pharmaceuticals from cyclodextrin/polymer formulations can be enhanced further. Comparing RM-β-CD and ML-β-CD formulated with PVP, the highest flow occurs at an 8% CD concentration. The flow was 30–100% greater than in solutions formulated without polymer. For RM-β-CD and CM-β-CD, the polymer predominantly influenced P_M/K_, but for ML-β-CD and HP-β-CD, M_1/2_ was impacted. The polymers cannot permeate the skin and are anticipated to influence M_1/2_ by enhancing the affinity of D∙CD for the aqueous diffusion layer [57].

In another study, saturated HC solutions were formulated in an aqueous buffer (pH 7.4) containing 0, 1, 5, and 10% HP-β-CD. The permeability and flux of the medicines through a PAMPA membrane at varying unstirred water layer (UWL) thicknesses were assessed. The more than two-fold differential in flux observed without HP-β-CD was eliminated with the addition of even 1% HP-β-CD to the donor phase. The incorporation of HP-β-CD into systems with greater UWL thicknesses markedly enhanced compound flow. The impact of HP-β-CD was correlated with its association constant (K_1:1_) with the model medications and diminished as the UWL thickness decreased to a specific minimal value. This indicates that the diffusion resistance of the UWL (R_Aq_) must exceed the resistance of the membrane (R_M_). In other terms, for HP-β-CD to exert an enhancing impact, R_Aq_ must significantly contribute to the overall barrier resistance. This indicates that hydrophilic cyclodextrins improve flow when the UWL resistance substantially affects the total barrier resistance [29].

The transdermal iontophoretic administration of HC solubilized in an aqueous solution of HP-β-CD was examined and compared with the chemical enhancement of co-solvent formulations. The passive absorption of HC through human cadaver skin was greater when administered by propylene glycol compared to solubilization in an aqueous solution of HP-β-CD. Nonetheless, the iontophoretic administration of the 1% HC-9% HP-β-CyD solution surpassed the passive delivery of the 1% hydrocortisone–propylene glycol formulation, despite the incorporation of oleic acid as a chemical enhancer. The iontophoretic administration of 1% HC with 3% or 15% HP-β-CD was inferior to that of the 9% HP-β-CD solution. The data indicate that free hydrocortisone, rather than its complexes, is primarily administered iontophoretically through the skin, while the HP-β-CD complex functions as a carrier to restore HC depletion in solution. HP-β-CD inhibits the formation of a skin reservoir for HC. Iontophoresis significantly enhances the transdermal distribution of HC compared to the chemical method when an adequate amount of HP-β-CD is incorporated to solubilize the HC [39].

#### 5.1.4. Drug Delivery Systems

Efficient methods for enhancing pharmacological qualities include the development of sophisticated dosage forms of CDs, such as the concurrent application of several CDs and an appropriate amalgamation of molecular encapsulation with alternative carrier materials [58]. The aim of one research was to examine chitosan microspheres as carriers for HC. Microspheres containing entrapped HC or a HC-CD combination were synthesized using the spray-drying method. The drug loading, release, and stability were examined [19]. The phase solubility diagram for the complex formation between HC and HP-β-CD was established. The solubility of HC (0.07 mg/mL) increased linearly with the concentration of HP-β-CD. The apparent stability constant (Ks) of the HP-β-CD complex was determined using the solubility diagram, presuming the formation of a 1:1 complex initially. The calculated apparent Ks was determined to be 466 M^−1^. The phase solubility investigation demonstrates the viability of producing an HC/HP-β-CD solid inclusion complex through the spray-drying of solubilized HC in a CD solution utilizing ethanol, which effectively dissolves HC and yields a clear solution upon mixing with aqueous HP-β-CD solution. The spray-drying technique can be utilized to generate complexes with high drug content (25% HC) and chitosan microspheres exhibiting favorable morphological attributes, narrow size distribution, and high encapsulation effectiveness for HC [19].

Recently, the electrospinning of nanofibrous webs containing APIs has proven to be a highly advantageous method for creating fast-dissolving drug delivery systems. Electrospinning may serve as an alternative to freeze drying, offering the potential for continuous production of solid nanofibrous formulations that incorporate APIs with exceptionally high encapsulation efficiency [59]. The electrospinning of nanofibers from aqueous solutions of cyclodextrin inclusion complexes containing API offers the benefit of utilizing solely water for the process, eliminating the necessity for a polymeric carrier and organic solvents. Furthermore, the electrospinning of API/CD inclusion complex systems devoid of any polymeric matrix would confer additional benefits for the advancement of rapid-dissolving oral-drug-delivery systems [60]. In one of the studies, HC was chosen as a model API for the electrospinning of polymer-free nanofibrous webs comprising a HC/CD inclusion complex, aimed at developing orally fast-dissolving drug-delivery systems. HC/HP-β-CD complexes were formulated in aqueous solutions with molar ratios of 1:1, 1:1.5, and 1:2 (HC/HP-β-CyD). Highly concentrated aqueous solutions of HP-β-CD (180%, *w*/*v*) were employed for HC/HP-β-CD systems (1:1, 1:1.5, and 1:2) to facilitate electrospinning without the incorporation of an extra polymer matrix. The turbidity of HC/HP-β-CD (1:1 and 1:1.5) aqueous solutions suggested the presence of uncomplexed HC crystals, while the aqueous solution of HC/HP-β-CD (1/2) was homogeneous, indicating complete water solubility of HC through inclusion complexation with HP-β-CD. Nevertheless, the electrospinning of HC/HP-β-CD systems (1:1, 1:1.5, and 1:2) effectively produced homogenous nanofibrous structures devoid of defects. Furthermore, the electrospinning method demonstrated high efficiency, resulting in the total preservation of HC without any loss, thereby producing HC/HP-β-CD nanofibers with initial molar ratios of 1:1, 1:1.5, and 1:2. The structural and thermal analysis of the HC/HP-β-CD nanofibers demonstrated that HC was entirely complexed with HP-β-CD and existed in an amorphous state within the HC/HP-β-CD (1:2) nanofibers, while uncomplexed crystalline HC was observed in the HC/HP-β-CD (1:1 and 1:1.5) nanofibers. Nonetheless, HC/HP-β-CD (1:1, 1:1.5, and 1:2) complex aqueous systems were electrospun into nanofibrous webs characterized by their free-standing and flexible properties. The HC/HP-β-CD (1:1, 1:1.5, and 1:2) nanofibrous webs exhibit rapid dissolution in water or upon contact with artificial saliva. The HC/HP-β-CD (1:2) nanofibrous web disintegrated more rapidly than the HC/HP-β-CD (1:1 and 1:1.5) nanofibrous webs, attributable to the complete inclusion complexation and the amorphous condition of HC in this sample. The findings indicated that polymer-free electrospun nanofibrous webs derived from HC/HP-β-CD may be highly suitable for rapid-dissolving oral medication delivery systems (Table 4) [20].

Solid lipid nanoparticles (SLNs) are prospective colloidal therapeutic systems capable of transporting lipophilic or hydrophilic APIs or diagnostics. The primary objective of another study [21] was to examine the integration of complexes between HC and two CDs, namely, β-CD and HP-β-CD, into SLN. The inclusion complexes of HC with β-CD and HP-β-CD were synthesized via the coprecipitation method, with a molar ratio of 1:2 for drug to β-CD.

An excess of HC was incorporated into a β-CD or HP-β-CD ethanol–water solution (30:70 *v*/*v*) and magnetically swirled for 72 h to yield the complexes. The residue was filtered, washed, and dried under vacuum [21]. 

The complexes of HC with HP-β-CD were produced by freeze-drying the filtered solutions to verify complex formation using the coprecipitation method. The SLN containing free HC exhibited a larger average diameter compared to those containing the HC and β-CD complex; also, the polydispersity values of these SLN dispersions were elevated, signifying a broader spread of the SLN population. HC integrated into SLN, whether as individual molecules or as complexes with two β-CDs, exhibited no crystalline structure and was instead disseminated inside the SLN in an amorphous state. The drug integrated into the solid lipid nanoparticles as complexes with β-CDs significantly delayed the drug’s release from the SLN [21].

Nanospheres and nanocapsules composed of amphiphilic β-CD, β-CD-C6, were assessed with a selection of steroid pharmaceuticals to ascertain the influence of drug physicochemical characteristics (such as partition coefficient, API:CD association constant K_1:1_, and aqueous solubility) on the loading and release dynamics of the nanoparticles. Inclusion complexes were generated between HC and β-CD-C6 by the co-lyophilization process and characterized using DSC analysis and FT-IR spectroscopy. HC exhibited its melting endotherm at 225 °C, which was absent in the thermogram of the co-lyophilizate HC/β-CD-C6 [22]. 

The findings suggest that the API is not present in a free amorphous state within the complex and is likely encapsulated within the CD cavity or long alkyl chains as the precise thermal behavior of β-CD-C6 is similarly observed in the complex. The loading procedure was the critical factor that influenced the drug release patterns from nanospheres [22].

The physicochemical features of the APIs did not significantly influence the in vitro release profiles. This affirms that drug release from colloidal carriers is contingent upon both the carrier type and the loading strategy employed. In contrast to nanospheres, the scenario for nanocapsules was notably distinct [22].

The release was considerably slower than anticipated and was extremely contingent upon the partition coefficient and aqueous solubility, with the release rate exhibiting an inverse correlation to the octanol–water partition coefficient [22].

Another publication outlines a technique for generating microcapsules of a hydrophilic, water-soluble CD complex that incorporates a lipophilic, water-insoluble API. The drug utilized in the sample was HC, and the complexing agent employed was HP-β-CD. This inquiry evaluated two methods: a phase separation approach and an emulsion–solvent evaporation method for the manufacture of microcapsules containing the hydrophilic and highly water-soluble HC/HP-β-CD complex. The lipophilic, water-insoluble HC was effectively microencapsulated using the phase separation process, yielding uniformly formed microcapsules. Nevertheless, when researchers attempted to employ this approach for microencapsulating the HC/HP-β-CD complex, the outcome was irregularly shaped clusters that were likely only partially coated with ethylcellulose. The compound seems to dissociate throughout the microencapsulation process. Uncontrolled and rapid HC release profiles were achieved from the “microencapsulated” HC/HP-β-CD complex. The release rate of HC from the “microencapsulated” HC/HP-β-CD complex was significantly more rapid than that from free (uncoated and uncomplexed) HC. The manufacture of microcapsules containing the HC/HP-β-CD complex by the emulsion–solvent evaporation approach was significantly more straightforward than the prior method. HP-β-CD and HC were dissolved in the dispersion phase during preparation, and the complex was produced upon evaporation. Consequently, lyophilization to create the solid complex was unnecessary. The product comprised uniformly formed spherical microparticles featuring a smooth surface. The release rate of HC from microcapsules composed only of the HC/HP-β-CD complex and ethylcellulose polymer is exceedingly sluggish. Approximately 30 percent of the API was released within the initial 150 min. The incorporation of a plasticizer markedly enhances the release rate, which can indeed be regulated by the quantity of plasticizer utilized in the microcapsule fabrication process. The findings indicate that although the dissolution of free HC was expedited by the introduction of a surfactant or the water-soluble complexing agent HP-β-CD, the release rate of HC from the microcapsules remains largely unchanged. The disintegration of uncoated HC/HP-β-CD complex occurred virtually instantaneously under identical conditions [23].

Hyaluronic acid (HA) hydrogels are appealing materials for biomedical applications due to their porosity, water absorption, biocompatibility, biodegradability, and resistance to non-specific cell adhesion [61]. A constraint of HA hydrogels is that the inclusion of bioactive pharmaceuticals may be hampered by the limited solubility of the drug within the hydrogel matrix. The incompatibility of hydrophobic APIs with the aqueous hydrogel environment may lead to drug crystallization, resulting in diminished hydrogel characteristics and unpredictable release kinetics. Furthermore, hydrophobic pharmaceuticals may ascend to the hydrogel’s surface during the drying process, leading to a rapid release [61,62]. The objective of the researchers was to create HA hydrogels that encapsulate drugs via hydrophobic interactions to enhance drug loading capacity. They functionalized photocrosslinked HA hydrogels with a methacryloyl derivative of β-CD. HA hydrogels modified with methacryloyl-β-CD monomer acquired the ability for inclusion complexation, significantly improving the absorption of the hydrophobic model drug, HC. The pre-incubation of the hydrogels with adamantane carboxylic acid (ACA) impeded HC absorption by competing for β-CD cavities. Moreover, the hydrogels of HA functionalized with α-CD monomer exhibited inefficiency in HC absorption due to the inadequacy of the α-CD cavity size for effective complexation [24].

The experiments validated that the HA-β-CD hydrogels with drug-binding properties can be further modified to provide HA-based biomaterials that incorporate drug delivery capabilities [24].

Over the past decade, numerous additive manufacturing technologies have been investigated to facilitate a more individualized patient treatment strategy. Inkjet printing is increasingly recognized in the advancement of customized oral dosage forms [63]. A study aimed to utilize the adaptability of inkjet printing to create flexible doses of drug-loaded orodispersible films that incorporated information in a data matrix format and to present specialized data matrix-generator software tailored for the healthcare sector. Hydrocortisone-loaded pharma-inks were developed and characterized according to their rheological characteristics and drug content. Various tactics were examined to enhance HC solubility, including the creation of β-CD complexes. The software autonomously adjusted the dimensions of the data matrix and determined the quantity of layers for printing. The development of β-CD complexes enhanced the medication quantity deposited in each layer. The enhancement was more pronounced in the pharmaceutical inks with a reduced 1,2-propylene glycol (PG) content. Solubility assessments were conducted on inclusion complexes at two distinct HC:β-CD mass ratios, 1:3 *w*/*w* (molar ratio 1:1) and 1:6 *w*/*w* (molar ratio 1:2), throughout varying incubation durations (1 day or 7 days). A higher concentration of CD (1:6 *w*/*w*) resulted in a more significant enhancement in solubility compared to 1:3 *w*/*w* and the absence of CD, for both 60:40 *v*/*v* and 30:70 *v*/*v* HC:β-CD ratios. The total quantity of HC in 25 mL of the β-CD-containing pharma-inks was 145.75 mg, 168.5 mg, and 185 mg for the PG:H_2_O ratios of 60:40 *v*/*v*, 1:3 *w*/*w*, and 1:6 *w*/*w* HC:β-CD complex pharma-inks, respectively. The total quantity of HC measured in 25 mL was 27.5 mg, 60 mg, and 75.25 mg for PG:H_2_O ratios of 30:70 *v*/*v*, 1:3 *w*/*w*, and 1:6 *w*/*w* HC:β-CD complexes, respectively. Consequently, the initial quantity of HC (300 mg) incorporated into the ink was not entirely encapsulated and was diminished in comparison to the PG:H_2_O 60:40 *v*/*v* pharmaceutical ink. The total quantity of HC encompasses both free HC (not complexed with β-CD) and complexed HC. The methanol employed as the mobile phase in HPLC analysis disrupted the HC:β-CD complexes, allowing for the accurate quantification of the total HC content in the pharma-inks (both dissolved HC and complexed HC) using the original HPLC methodology [25].

Although a higher CD ratio yields somewhat increased solubility, the enhancement achieved was insufficient relative to the resource expenditure required to make the 1:6 *w*/*w* HC:β-CD complex [25]. Consequently, the ink comprising HC:β-CD (1:6 *w*/*w*) was eliminated. The solubility of HC at various incubation durations was examined utilizing the identical ratio of HC:β-CD. The solubility increased marginally with extended incubation period, measuring 9.85 mg/mL after 7 days compared to 6.74 mg/mL after 1 day. Based on the results, it can be concluded that for the tested durations of 1 and 7 days, shorter incubation times were more effective as the primary goal was to produce on-demand films [25].

Orodispersible films incorporating flexible and low doses of personalized HC were effectively produced, and the creation of a code generator program tailored for medical applications offered a supplementary, innovative, and transformative benefit to the safety and accessibility of personalized medicine [25].

The impact of HP-β-CD on the solubility of drugs and their release from suppository bases was examined for HC, hydrocortisone acetate (HCA), and prednisolone acetate (PNA). Four distinct suppository formulations were developed for each drug using the fusion process, incorporating 2.5% drug in either a PEG 1000/PEG 3350 (1:1) base or a cocoa butter basis, with or without HP-β-CD at a 1:1 molar ratio to the drug. A uniform mixture of a suppository melt was poured to slightly exceed the capacity of a VanKel Enhancer cell, which had been previously calibrated to hold a volume corresponding to 1 mL of water. The mixture was let to solidify at ambient temperature, and the surplus that overflowed the cell was eliminated. A suitable synthetic membrane was utilized and affixed to cover the upper surface of the solidified mixture. The observed water solubility of all examined steroids was significantly enhanced by HP-β-CD. The enhancement of solubility by HP-β-CD augmented both the rate and amount of drug release from the water-soluble base while having no impact on drug release from the oil-soluble base. This investigation does not allow for a conclusion regarding the impact of the oil/water partition coefficient on drug release from either type of base [26].

#### 5.1.5. Ophthalmic Applications

Another research study demonstrated that β-CD tetradecasulfate can enhance the antiangiogenic efficacy of both natural and synthetic corticosteroids and that both angiostatic medication combinations are efficacious when administered topically to the cornea. Utilizing ideal ratios of steroid and CD resulted in a reduction in neovascularization to 13%, 26%, and 28% of untreated controls for the three hormones (HC, tetrahydrocortisol-S, and 6-α-fluoro-17,21-dihydroxy-16-β-methyl-pregna-4,9,(ll)-diene-3,20-dione), respectively. For HC, the optimal steroid–cyclodextrin ratio was 1:2 [36].

Steroids prepared as suspensions or solutions have low corneal permeability, mostly due to the low aqueous solubility of the steroid in suspensions or the inadequate partitioning of the hydrophilic steroid derivative into the lipophilic epithelium in the case of water-soluble salts [64]. As a result, the ocular bioavailability of topically applied steroids is expected to be below 0.5% [65].

The impact of HP-β-CD on the aqueous solubility and chemical stability of HC was examined with the objective of developing a stable topical ophthalmic solution of HC. The ocular bioavailability after topical treatment to rabbits of the aqueous formulation of HC was compared to that of a suspension formulation with an equivalent HC content. The aqueous solubility of HC significantly increased with the addition of HP-β-CD due to the creation of a soluble 1:1 inclusion complex. The apparent association constant of the HC/HP-β-CD complex, as measured by phase-solubility analysis, was estimated to be 0.636 mM^−1^. Complexation with HP-β-CD enhanced the chemical stability of HC, resulting in a reduction in the pseudo first-order rate constants of decomposition to 6.73 × 10^−3^ and 0.90 × 10^−3^ h^−1^. The ocular bioavailability after topical treatment to rabbits of a tritium-labeled 1% HC solution formulation of the HC/HP-β-CD complex was inferior to that of a 1% suspension formulation. A notable decrease (*p* < 0.05) of 25 to 40% was seen in the cornea, aqueous humor, iris, and sclera [17]. Another researcher found that a 1% *w*/*v* HC formulation as an HC/HP-β-CD solution with 69.7 mM CD yields an approximate 60% enhancement in ocular bioavailability relative to a suspension in New Zealand White rabbits. In vitro, the corneal permeability of HC is linearly proportional to [HCf] (the concentration of free HC in solution) and remains consistent whether produced as a suspension or HC/HP-β-CD solution, given that HCf ≤ S_0_ (S_0_ is the solubility of the neutral form of a compound). This indicates that the rise in ocular bioavailability in vivo is not attributable to an improvement in corneal permeability. The linear correlation between permeability and [HCf] may elucidate the reduced in vivo ocular bioavailability of HC when combined with 90 mM HP-β-CD, as observed by Davies et al. [37].

The permeation-enhancing impact of γ-CD on saturated HC medication solutions was assessed using cellophane and fused cellulose–octanol membranes within a typical Franz diffusion cell method. The enhancement of drug permeation, resulting from the addition of γ-CD, was defined by the flux ratio (J_R_), defined as the rate of the flux (J) determined in the saturated drug solution with and without γ-CD. The J_R_ values, being significantly larger than 1, indicate that γ-CD enhances drug permeability (Table 5) [18].

Commercial gelatin is classified into two categories, commonly referred to as type A and type B. Gelatin type A is sourced from acid-processed collagen, whereas type B is produced through alkaline collagen treatment, leading to distinct isoelectric values of 7–9 for type A and 4–5 for type B. Gelatin nanoparticles encapsulating hydrocortisone as a model API were synthesized by a desolvation process. Particles were produced at pH 4 and 6. The impact of various preparation settings on particle characteristics was examined. The zeta potential values of gelatin type A and gelatin type B particles were compared. At pH 4, both gelatin types A and B possess a positive charge. At a pH 6, gelatin type A retains a positive net charge, but gelatin type B exhibits a negative charge. The drug encapsulation was roughly 30–40%. A sustained release was found in comparison to the aqueous drug solutions. The release data indicate a variation in release rate based on the type of CD utilized. After 5 h, over 60% of the HC was located in the acceptor compartment for the reference solutions of HC complexed with HP-β-CD and Cat-CD (2-hydroxy-3-trimethyl-ammoniopropyl cyclodextrin). The particle preparations exhibited a diminished release rate, achieving just 30–40% after 5 h. The preparations utilizing HP-β-CD to complex HC exhibited the slowest release, with less than 30% after 300 min. The utilization of cationic CD resulted in over 30% of the API being located in the acceptor compartment for particles formulated with gelatin type B and exceeded 40% for those with gelatin type A nanoparticles [38].

The comparative analysis of the results presented in this section shows that the complexation of HC with various CDs has a long, almost half-century history. While it has been proven that HC can form stable (K > 10^3^) complexes with multiple CDs, both native and substituted, usually the β-CD is being used as a host molecule. However, some studies show that more stable complexes of this API are being formed with the larger CDs, namely, γ-CD and its derivatives. There is an agreement that the complexation of HC increases substantially its solubility, regardless of the CD used for that purpose. Additionally, increased permeability has been observed in multiple studies. However, some discrepancies exist on the molar ratios of those complexes, i.e., while most of the reviewed studies point out the 1:1 stoichiometry of HC/β-CD, other ones indicate that 1:2 is the proper one. More recent works suggest that both types of complexes can be formed in aqueous solutions, with their K depending highly on temperature, pH, and the presence of the other substances. It is also worth noticing the large number of works presenting the results of the studies on the novel dosage forms, consisting of HC/CD complexes, particularly for ophthalmic application.

### 5.2. Cortisone and Cortisone Acetate

#### 5.2.1. Complex Preparation and Structural Studies

Similar experiments to the ones conducted for HC/β-CD, described in Section 5.1.1, have been performed by the same group of authors, this time for CO [14]. The equilibrium solubility investigations have revealed B-type behavior in the CO/β-CD systems, as indicated by the solubility phase diagrams. The solubility phase diagram exhibits a notable rise in the overall concentration of steroids due to complexation in its early section. The initial rise in the solubility of steroids is significantly higher compared to the solubility found in the later part of the solubility phase diagram at high concentrations of β-CD. The solubility reaches a level similar to that of the pure 1:2 steroid complex when the concentration of β-CD exceeds 20 mM. The solid phase from this location was analyzed using mass spectra and chemical analysis of a pure complex. The results suggest that the complexes in the solid state have a stoichiometric ratio of 1:2. The FAB mass spectra revealed the presence of both 1:1 and 1:2 complex molecular ions among the mass fragments collected [14].

**Figure 11:**
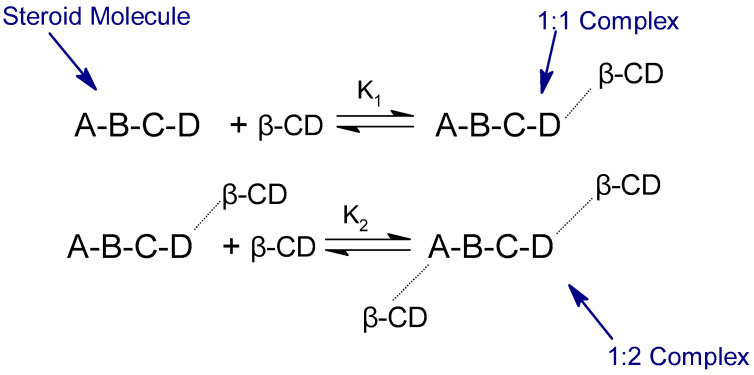
Schematic of a complexation process between a steroid molecule and two β-CD molecules.

The relative sizes of K_1_ and K_2_ indicated that the cortisone initially forms an inclusion complex with one molecule of β-CD, which seems to be a more preferred procedure compared to the formation of a 1:1 complex with the second molecule of β-CD. Regarding cortisone, in contrary to HC, the results indicated that the second interaction of the 1:1 complex with the β-CD molecule is a preferred mechanism compared to the initial association. The unusual behavior seen may be attributed to the creation of a 1:1 complex, leading to the disruption of the intramolecular hydrogen bonding between the ketone group at position 11 and the main hydroxyl group of the side chain at position 17 in CO. The presence of the unbound ketone group can enhance the lipophilicity of the 1:1 complex and facilitate its hydrophobic association with the second β-CD. It is important to acknowledge that while K_2_ is larger than K_1_ for CO, the computation of both K values remains accurate because the premise that K_2_·[L] < 1 still holds true. The K_2_ values obtained from the dissociation of the pure complex are in good agreement with the K_2_ values derived based on the proposed model (Figure 11) [14].

The FTIR spectrum of the CO/β-CD complex showed a displacement in the wavenumber of the carbonyl group at position 3 by approximately 24 cm^−1^. In contrast, those changes were not observed in the spectra of the physical mixture. This suggests that the interaction between steroids and β-CD in a water-based solution is influenced by the side chains of the steroids [14].

#### 5.2.2. Biotransformation

Microorganisms are frequently used to perform specific reaction steps in the manufacture of different steroidal medicines and pharmacological intermediates [66]. Nevertheless, the significant toxicity of organic solvents to cells, leading to their limited tolerance by microorganisms, remains a constraining factor in the process [67]. As a result, CDs have been extensively employed to create host–guest complexes in order to enhance the reaction rate and extent of conversion in numerous steroid biotransformations. Specifically, the enhancement of solubility, stability, and bioavailability has been extensively utilized to facilitate and improve microbe-mediated transformations of hydrophobic chemical molecules [68]. 

A thorough and methodical examination was conducted on the process of converting cortisone acetate (CA) to prednisone acetate (PA) by Arthrobacter simplex TCCC 11037. This inquiry involved the use of both unaltered and altered forms of CDs [15]. The investigation of the inclusion complexes between CDs and CA was conducted using phase solubility, 2D NMR spectroscopy, and DSC. The variation in structure of CDs led to differences in stoichiometry among the complexes. The CA/RM-β-CD, CA/SBE-β-CD, and CA/HP-β-CD complexes had a 1:1 ratio, while CA/β-CD formed both 1:1 and 1:2 complexes. The 1:2 complex reduced the soluble CA concentration and hindered the dissociation of β-CD/CA in an aqueous solution. The solubility of CA increased in the following order: RM-β-CD > SBE-β-CD > HP-β-CD > β-CD. CA/RM-β-CD, CA/SBE-β-CD, and CA/HP-β-CD/CA showed a greater biotransformation rate as compared to native β-CD [15]. 

**Figure 12:**
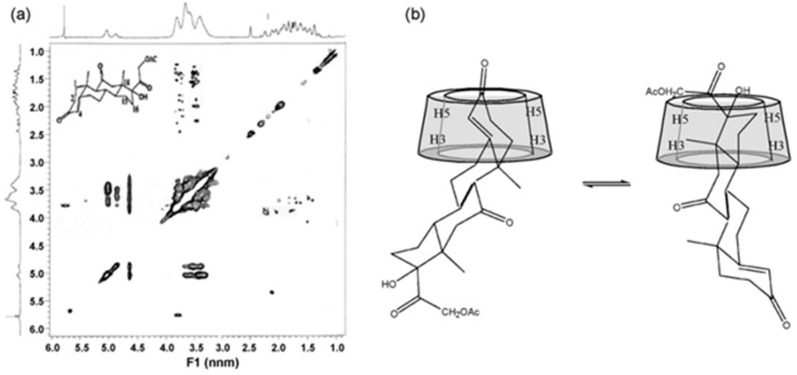
(**a**) ROESY spectrum of the inclusion of β-CD-CA in D_2_O at 25 °C with a mixing time of 400 nm. (**b**) Possible structure of the inclusion complex of β-CD-CA based on the ^1^H ROESY NMR experiment. Reprinted with permission from [15].

Recently, 2D NMR spectroscopy has emerged as a significant technique for studying the structure of host–guest inclusion complexes in CDs. In one of the variants of this method, the Nuclear Overhauser Effect (NOE) correlations between the protons of the guest and the inner protons of the CD cavity (H3 and H5) are measured. By analyzing the relative intensity of these cross-peaks, it is possible to determine the orientation of the guest molecule inside the CD cavity. Figure 12a demonstrates that the ROESY spectrum exhibited evident NOE cross-peaks between the H5 protons of β-CD and the H18/H21 protons of CA, as well as between the H3 of β-CD and the H15 proton. Additionally, cross-peaks were observed between H3/H5 of CD and the H16 protons of CA. These findings clearly indicate that the D ring in CA was encompassed within the hydrophobic cavity of β-CD. Furthermore, there are distinct correlations observed between the H5 protons of β-CD and the H1/H2 protons of CA, as well as between the H3/H5 of β-CD and the H4 proton of CA. These correlations indicate that the A ring in CA was enclosed within the hydrophobic cavity of β-CD. Based on the ROESY data, the authors could infer a potential binding mechanism of the CA-CD complex, as shown in Figure 12b. These experiments provide additional support for a subsequent phase-solubility investigation that demonstrated that CA has the ability to form a 1:2 inclusion complex with β-CD [15].

Another study has also verified that HP-β-CD is a potent solubilizer for enhancing the solubility and dissolution rate of CA in the aqueous phase during the enzymatic biotransformation process. By employing HP-β-CD (20% *w*/*v*), the aqueous solubility of CA was enhanced from 0.039 to 7.382 g L^−1^ at 32 °C. HP-β-CD exhibited a much greater solubilization impact compared to dimethylformamide (DMF) and ethanol. The inclusion of CA into HP-β-CD considerably enhanced its disintegration rate. The enzymatic stability of Δ1-dehydrogenase from *Arthrobacter simplex* TCCC 11037 remained unaffected by the rising concentrations of HP-β-CD. However, the presence of organic cosolvents, specifically DMF and ethanol, had a negative impact on the enzymatic stability. The inhibitory effect on activity caused by HP-β-CD was less pronounced compared to ethanol and DMF. The inactivation constants for ethanol, DMF, and HP-β-CD were 5.832, 4.541, and 1.216, respectively. The activation energy (Ea) followed the sequence: HP-β-CD (55.1 kJ mol^−1^) > ethanol (39.9 kJ mol^−1^) > DMF (37.1 kJ mol^−1^). A less amount of HP-β-CD was required to achieve a specified solubility, hence minimizing the inhibitory effect of the additive. HP-β-CD had the least impact on both enzymatic stability and thermal stability when compared to DMF and ethanol. While the presence of HP-β-CD led to a reduction in enzymatic activity, the extent of activity inhibition was significantly lower, compared to an equivalent amount of DMF and ethanol. Thus, HP-β-CD effectively enhanced the solubility of the substrate while simultaneously reducing the loss of activity and stability of the biocatalysts. Furthermore, it has been demonstrated that HP-β-CD has no impact on pH. As a result, there is no need for pH monitoring or correction, leading to a simplification of the application process. In addition, HP-β-CD is non-toxic and does not react with microorganisms, unlike organic cosolvents. This makes it a promising candidate for replacing organic solvents in the domains of biocatalysis and biotransformation [16].

#### 5.2.3. Application of the Complexes in the Capacitive Determination in Biofluids

Contrary to other sepsis biomarkers with a brief duration of effectiveness, cortisol is a reliable and enduring sepsis biomarker [69]. Nevertheless, cortisol levels exhibit rapid fluctuations, and assessing blood cortisol concentrations necessitates a blood test, which has the potential to elevate stress hormone levels. Better information can, therefore, be obtained by measuring cortisol in noninvasive relevant biofluids like saliva and urine [70].

The objectives of one of the papers were twofold: firstly, to create and analyze two polypropylene glycol (PPG) surfaces in order to identify the most durable and reusable β-CD biosensor and, secondly, to assess the effectiveness of the biosensor in measuring cortisol levels in biofluids (specifically urine and saliva) using nonfaradaic electrochemical impedance spectroscopy (EIS) [13]. 

The choice of those two excipients, namely, β-CD and PPG, was not random. First, β-CD and cortisol have the ability to form host/guest inclusion complexes [39]. Furthermore, the hydroxyl groups of β-CD have the ability to form hydrogen bonds with polymer materials, specifically PPG derivatives [71]. Additionally, β-CD can form inclusion complexes with PPG when the molecular weight of PPG is lower than 2000. It is believed that in such complexes for every three propylene glycol units, there is one β-CD molecule. First the researchers validated this estimation using 1H NMR, the technique that has been also employed to verify the binding of β-CD and cortisol. The data indicated that there was a binding ratio of 1:2 for cortisol/β-CD complexes. From the stoichiometric point of view, in the absence of PPG, when just cortisol and β-CD are present in the solution, the ratio of cortisol to CD is 0.5:1. Similarly, in the absence of cortisol in the PPG and β-CD solution, the ratio of PG to CD is 3:1. Nevertheless, in the presence of CD, both cortisol and PPG interact. Consequently, the addition of cortisol to the β-CD and PPG solution reduces the interaction between PG units and β-CD. In such systems, each β-CD molecule interacts with just 1.4 PG units instead of the previous 3 PG units.

This can be explained as the remaining β-CD molecules engage in interactions with cortisol. Similarly, the addition of PPG to the β-CD and cortisol solution results in a reduced interaction between cortisol units and β-CD. Specifically, each β-CD molecule now interacts with 0.22 cortisol molecules instead of the previous 0.5 cortisol molecules. Similarly, this is explained by the β-CD interaction with both PPG and cortisol, while the remaining β-CD molecules engage with PG units [13].

**Figure 13:**
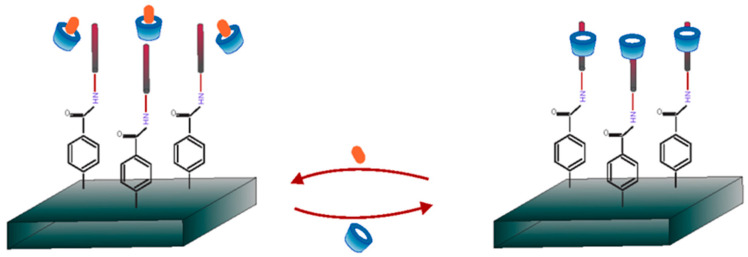
Demonstration of GC-carboxyphenyl-PPG:βCD surface for reusable sensing of cortisol: schematic that demonstrates (**left**) release of βCD from PPG to interact with cortisol and (**right**) surface regeneration by βCD reloading. Reprinted with permission from [13]. Copyright 2009 American Chemical Society.

In the subsequent study, the scientists utilized the technique described above to create a cortisol biosensor using competitive interactions between β-CD, PPG, and cortisol. Furthermore, the stability of the biosensor was enhanced by substituting the gold−thiol bonds with the more robust C–C triple bonds using diazonium salt chemistry. Due to increased stability, the GC-carboxyphenylPPG:βCD complex could be regenerated and reused for a maximum of 10 cycles (Figure 13). Ultimately, the biosensor was utilized to detect cortisol in human urine and saliva with high sensitivity and specificity [13].

### 5.3. Prednisolone

#### 5.3.1. Complexes with Different Cyclodextrins

In one of the very first studies on the prednisolone/CD complexes, the authors compared the solubility of prednisolone (PDL) with a wide variety of both native cyclodextrins and their derivatives, including α-, β-, and γ-CD, glucosylated, maltosylated, HP- and SBE-β-CD, and some unique SBE-γ-CD derivatives. The arrangement of the inclusion complexes formed by β-CD and PDL was determined using two-dimensional (2D) NMR spectroscopy [40].

Phase-solubility diagrams of PDL with various CDs at 25 °C were obtained. The intrinsic aqueous solubility of prednisolone was 391 ± 8 mg/L. All CDs, except for γ-CD, displayed A_L_-type diagrams within the specified concentration range. The γ-CD demonstrated a B_S_-type diagram that showed a consistent linear growth until reaching a concentration of 0.0125 M. Subsequently, a plateau was attained, suggesting that the inclusion complex had a restricted solubility. The A_L_-type diagrams and the primary linear slope on the B_S_ diagrams exhibited a slope below one, suggesting the likely creation of 1:1 complexes in an aqueous solution [40].

The binding site of PDL is predominantly located in ring A, as evidenced by its extensive penetration into the CD cavity. This is supported by the intensity of cross-peaks to H5 and the presence of cross-peaks even to H6. Two more interaction sites can be discerned at rings C, specifically H11 and H12, and at ring D, specifically H15, H16, and H21 of the hydroxymethyl-carbonyl side chain. The latter interaction has a significant lack of strength. In both instances, the steroid does not deeply penetrate the CD cavity [40]. 

The association constants of the prednisolone complexes with (SBE)_9M_-γ-CD and (SBE)_4M_-γ-CD were substantially greater than those of the underivatized γ-CD [40].

The carboxyl group of prednisolone 21-hemisuccinate was conjugated to a hydroxyl group of α-, β-, and γ-CD utilizing the coupling agent carbonyldiimidazole. The direct coupling generated prednisolone-conjugated cyclodextrins, wherein the API is preferentially attached to one of the secondary hydroxyl groups via an ester bond. The water solubility (>50% *w*/*v* at 25 °C) of these conjugates significantly exceeded that of prednisolone and its 21-hemisuccinate. Prednisolone was gradually released from the conjugate: the percentages of PDL and its hemisuccinates released from the α-, β-, and γ-CD conjugates were 49%, 57%, and 85%, respectively, over 24 h. The suggested release mechanism involved two rapid acyl migrations between the 2- and 3-hydroxyl groups of cyclodextrins and the 21- and 17-hydroxyl groups of PDL. The gradual release of PDL from the ester conjugates markedly differed from the rapid release of the PDL amide conjugation previously documented. The authors concluded that prednisolone-appended cyclodextrin conjugates may serve as an orally administered delayed-release and/or colon-specific prodrug due to their comparatively slow and/or site-specific-release characteristics [46].

#### 5.3.2. General Cyclodextrin Studies

The impact of β- and γ-CDs on the pharmacokinetics of PDL following intravenous or intramuscular injection in rabbits was examined. The serum concentrations of PDL following intravenous delivery were minimally influenced by the two CDs. This may have resulted from the fast dissociation of the complexes in the extensive amount of bodily fluids. Conversely, the serum concentrations of prednisolone following the intramuscular injection of the CD as a suspension were markedly elevated, compared to those of the PDL administered alone. The mean residence durations of both CD complexes were shorter than those of PDL alone. Conversely, there was minimal or no variation in pharmacokinetic characteristics following intramuscular administration of the drug as a solution, with the exception of the length of time to reach the maximum serum levels (T_max_) between PDL and its CD complexes. The increased bioavailability of prednisolone following intramuscular delivery as a suspension may result from the fast breakdown of its CD complexes [47].

The reaction of α-amino-ω-methoxypoly(ethylene glycol) [M = 5000] or star α-amino-poly(ethylene glycol) [M = 20,000] with hemiesters of prednisolone dicarboxylic acids (succinic, glutaric, adipic, and phthalic acid) has been employed to synthesize the respective conjugates. The rate of the esterase-catalyzed hydrolysis of the conjugates is influenced by the molecular weight of poly(ethylene glycol) and the length of the linker connecting PDL to poly(ethylene glycol) (t_1/2_ ~ 5–0.5 h). The enzymatic hydrolysis occurs more swiftly in conjugates containing linkers produced from adipic and phthalic acids. The synthesized conjugates created polypseudorotaxanes with α-CD, characterized by 2D NOESY NMR spectra, PXRD patterns, and, in one instance, STM microscopy. The enzymatic release of the polypseudorotaxane with a linker produced from adipic acid occurs approximately five times slower than the release rate of PDL from the equivalent conjugate. The prednisolone release rate from the carrier can be regulated by three factors: the nature of the linker connecting the polymeric carrier and PDL, the molecular weight of poly(ethylene glycol), and the development of complexes with α-CD. The synthesized polypseudorotaxanes signify innovative transport systems designed for the tailored release of prednisolone in transplanted liver tissue [72]. 

In another work, the acylation of prednisolone 20-hydrazone with star poly(ethylene glycol) tetracarboxylic acid (M = 20,000) has been employed to synthesize the appropriate pH-sensitive conjugate. This conjugate forms a polypseudorotaxane with α-cyclodextrin, characterized by 1H NMR spectra, powder X-ray diffraction patterns, and STM imaging. The rate of acid-catalyzed hydrolysis of the compound was examined in vitro using model media consisting of hydrochloric acid solutions and phosphate and acetate buffers (pH 2–5.8). The acid-catalyzed hydrolysis (at pH 2) of the polypseudorotaxane was approximately 3.5 times slower than that of the original compound. After one hour in this medium, 86% of the covalently bonded prednisolone remained unaltered. The synthesized polypseudorotaxane serves as a potential oral delivery method for prednisolone, featuring a pH-sensitive linker that enables delayed acid-catalyzed hydrolysis, facilitated by molecular protection through α-cyclodextrin [73].

The solubility and aerosolization characteristics of leucine-coated inhalable powders containing HP-β-CD complexed with PDL have been investigated. The dry particles were concurrently generated and coated with nanoscale L-leucine crystals via an aerosol flow reactor technique. The aerosolization efficacy of carrier-free powders was examined utilizing Easyhaler^®^ and Twister™ at pressure decreases of 2 and 4 kPa across the inhalers. The drug-penetration characteristics of the formulations were evaluated using a Calu-3 cell monolayer. Toxicity and the production of reactive oxygen species (ROS) were evaluated using Calu-3 and A549 cell lines. The HP-β-CD in the powders improved the dissolution kinetics of PDL. The fine particle fractions ranged from 52% to 70% of the emitted dosages, demonstrating good repeatability with a coefficient of variation between 0.9 and 0.17. Furthermore, HP-β-CD enhanced the permeability of PRE. The powders exhibited no statistically significant toxicity or production of ROS in the evaluated cell lines [74].

#### 5.3.3. Oral Delivery

One of the first studies focused on the oral delivery of complexes composed of PDL and β-CD investigated the dissolution in water and permeation through a cellophane membrane. In addition, a crossover bioavailability study was conducted on adult male volunteers, administering lower doses of PDL tablets and assessing plasma levels using radioimmunoassay. According to the dissolution profiles, it was certain that the complexed form of prednisolone dissolved much more rapidly than prednisolone itself. It was observed that the β-CD complex had a higher net amount of PDL penetrated into the receptor cell, especially when the dissolution rate was faster. However, the penetration rate of the complex seemed to be significantly lower than what was anticipated based on the dissolution profiles. The limited permeability of the large complex might be the cause, as the primary mechanism for permeation through a cellophane membrane is mostly controlled by the size of the pores. On the other hand, the complex caused a swift emergence of PDL in the bloodstream, with the concentration peaking at 0.54 μg/mL after 0.8 h of treatment, and then rapidly declining. The area under the curve (AUC), which is integral of the concentration of a drug in blood plasma as a function of time, of the combination up to 8 h was approximately 1.2 times greater than that of prednisolone alone. It is worth mentioning that the rise in AUC was quite minimal despite the fast-dissolving nature of the β-CD complex. This could be attributed to the high stability constant (3600 M^−1^) of the prednisolone-β-CD complex, as previously reported. This high stability constant may lead to a reduced release of free medication, limiting its availability for absorption in the gastrointestinal tract [42].

A porosity-regulated osmotic pump tablet is one that is covered by a semipermeable membrane holding substances that can be dissolved and released. In this formulation, the drug is released from the OPT (outer protective layer) due to hydrostatic pressure, which is generated by the dissolving of pore formers present in the membrane. These pore formers create pores through which the API molecule is delivered once it dissolves. The hydrostatic pressure is generated by an osmotic agent, either the API itself or a component of the tablet, once water is absorbed through the semipermeable membrane. This technique is mostly suitable for APIs that have a high solubility in water. Due to the slow dissolution of weakly water-soluble APIs, it is not feasible to achieve complete delivery of pharmaceuticals with low solubility qualities using such devices. This issue can be resolved by including (SBE)_7m_-β-CD or Captisol^TM^, which serve the dual purpose of solubilizing and acting as an osmotic agent [75].

Several studies have been designed to assess the effectiveness of the controlled-porosity osmotic pump tablet for HTC delivery. In one of them, the absorption of PDL was assessed in male beagle dogs using in vivo methods. Incomplete release was observed when HP-β-CD and a sugar combination of lactose and fructose were used. The release of PDL from the osmotic pump tablet with (SBE)_7m_-β-CD and the sugar formulation primarily exhibited zero-order release characteristics. However, the pill with HP-β-CD demonstrated clear first-order release characteristics. A research study conducted in dogs showed a strong correlation with the release patterns observed in laboratory tests using the dissolving method outlined in the Japanese Pharmacopoeia [48]. Advancements in this technology have resulted in ongoing studies regarding the viability of a pellet formulation. In other research, the improved solubility of PDL was achieved by using (SBE)_7M_-β-CD, which forms a 1:1 complex with this API. The binding constant of this complex was determined to be 1691 ± 98 M^−1^ using a UV method at 25 °C [43]. Initial investigations were carried out to ascertain the upper limit of CD that could be accommodated without encountering any issues (such as the formation of a viscous wet mass) during the process of pelletization. It was discovered that the formulation could accommodate a maximum of 40% of (SBE)_7M_-β-CD. When more than 40% of the wet mass was utilized, it was not possible to extrude and spheronize it. The quantity of CD was subsequently set at a constant rate of 35% for all subsequent tests. Due to the wet granulation process, the physical state of PDL was more intricate than anticipated. Specifically, the technology used for formulation was expected to alter the physical state of PDL during processing, which includes the creation of complexes with the water-soluble CD. The addition of CD to the control formulation with lactose resulted in a notable enhancement in the release of PDL from the CP-OPPs. Among the three different molar ratios of PDL to CD, the formulation with a molar ratio of 3:1 exhibited the most comprehensive release of PDL. This was followed by the formulations with molar ratios of 1:2 and 1:1. However, the release of CD as a percentage was comparable across all three formulations. The XRPD confirmed the amorphous state of the freeze-dried material. The physical combination powder exhibited distinct crystalline peaks for PDL; however, the freeze-dried powder did not exhibit similar peaks, likely due to the loss of crystallinity of PDL when it forms a complex with the amorphous CD [43].

Concluding, this kind of formulation offers numerous advantages compared to tablets, such as greater flexibility in designing and developing dosage forms, as well as enhanced safety and effectiveness in drug delivery. These benefits include reduced fluctuations in drug concentration in the blood, decreased side effects, minimal variability between individuals and within the same individual, and a lower risk of the sudden release of a large dose [76].

#### 5.3.4. Ocular Delivery

Barriers, such as eye blinking and lachrymal secretion, limit the effectiveness of topical ocular medication delivery, leading to limited bioavailability. The resolution of this issue poses a significant obstacle in the area of research and development since it is important to address it due to the fact that topically administered formulations exhibit the greatest level of patient compliance in the management of ocular conditions [77]. The drug’s transcorneal penetration is crucial for achieving maximum efficacy. In order to achieve the most effective transcorneal penetration, it is necessary to maintain a proper balance between the hydrophilicity and lipophilicity of both the medicine and the vehicle [78]. Glucocorticoid derivatives, such as prednisolone, are commonly employed in ophthalmic surgery to prevent postoperative inflammation. To enhance efficiency on the eye surface, one method is to dissolve the API in an aqueous solution. This ensures that the medicine is present in the correct concentration near the cornea’s epithelium [56].

One of the studies aimed to create a new eye drop formulation that contains PDL and can dissolve in water. The goal was to develop a formulation that only needs to be applied infrequently while still maintaining microbiological stability and acceptable physiological properties such as surface tension, pH, and osmolality. This was achieved by using a CD inclusion complex, as well as a preservative and mucoadhesive substances called zinc hyaluronate (ZnHA) and zinc gluconate (ZnGlu) [49].

**Figure 14:**
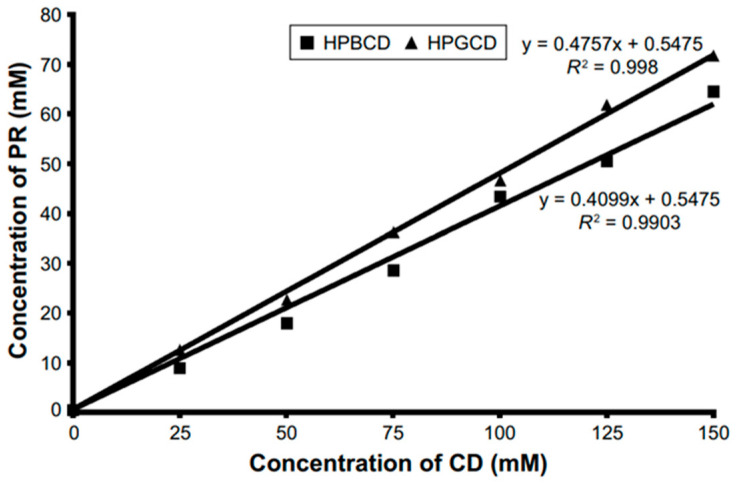
Phase solubility diagrams of prednisolone (called PR in this figure) in aqueous HP-β-CD and HP-γ-CD. Reprinted with permission from [49], licensed under CC BY 3.0.

The solubility of PDL exhibited a linear rise with the augmentation of HP-β-CD or HP-γ-CD concentration. Figure 14 illustrated Higuchi A_L_-type for both CDs, indicating the creation of 1:1 complexes. The apparent stability constant of the HP-β-CD complex is 1286.4 M^−1^, while the stability constant of the PDL/HP-γ-CD complex was measured to be 1778.5 M^−1^ [49].

The examination of the diffusion of PDL over the dialysis membrane found that the maximum penetration of API under in vitro conditions occurred with 5 mM of HP-β-CD and 4 mM of HP-γ-CD. The inclusion of ZnHA and ZnGlu did not impact the penetrating properties. The viscosity measurement indicated that the products containing 5-mM HP-β-CD and 4-mM HP-γ-CD had the lowest viscosity values, which satisfied the requirements of the EP. The study revealed that the concentration of CDs does not impact the surface tension of the eye drops. Furthermore, these values are considered optimal when compared to other ophthalmic products that have been previously studied [49].

The researchers furthered their investigation by examining the cytotoxicity and API permeability of the formulation using an in vitro human corneal cell line and an ex vivo porcine cornea model [79]. The toxicity was assessed using impedance measurement following the administration of various formulations. Based on the estimated normalized cell index, samples containing ZnHA-ZnGlu were found to be not toxic. However, samples containing benzalkonium chloride (BK) had significantly lower values, indicating a toxic effect on the HCE-T cells. The immunohistochemistry analysis also confirmed the toxicity of formulations containing BK. The cells treated with BK-containing formulations exhibited significant morphological alterations in the E-cadherin junctional protein [79].

No morphological changes or harmful effects were observed on the cell culture when using target eye drops containing ZnHAZnGlu. The ZnHAZnGlu combination can be considered a non-toxic alternative preservation system that is well-tolerated by HCE-T cells at the applied concentration [79].

In addition, the permeability of PDL was evaluated on the HCE-T model using various formulations. Simulating the absorption across the corneal barrier can be achieved by evaluating the substances on cell culture. The solutions containing PDL-CD complexes and the target formulations containing PDL-CD-ZnHA-ZnGlu were compared with a sample that included the same quantity of PDL in suspension form. The electric resistance was measured and found to validate the integrity of the cell layers. The permeability exhibited a substantial increase in samples containing the dissolved PDL-CD-ZnHA-ZnGlu complex as opposed to the suspension. Due to the greater concentration of dissolved API in PDL-CD solutions, particularly towards the corneal epithelial cell layer, faster drug absorption into the epithelial layer is anticipated [79].

#### 5.3.5. Liposomal and Nanofiber Delivery Systems

The inclusion complexes of prednisolone with β-CD and HP-β-CD were synthesized using the solvation method and characterized using DSC, PXRD, and FTIR. Egg phosphatidylcholine (PC) liposomes containing PDL, either as a standalone drug or as an inclusion complex, were synthesized via the dehydration–rehydration process, and both drug entrapment and drug release were assessed for all generated liposome variants. The maximum PDL entrapment value (80% of the initial amount) was attained for PC/cholesterol liposomes when the PDL/HP-β-CD (1:2, mol/mol) complex was encapsulated. The leaking of vesicle-encapsulated 5,6-carboxyfluorescein (CF) served as an indicator of vesicle membrane integrity. The experimental results indicated that liposomes encapsulating PDL/β-CD complexes exhibit markedly reduced stability, in terms of membrane integrity, compared to liposomes with identical lipid compositions that contain either the plain drug or, in certain instances, non-drug incorporating liposomes used for comparative analysis. Notably, liposomes encapsulating PDL/HP-β-CD complexes have minimal first CF latency values, suggesting that CF leakage occurs at a significantly high start velocity. Interactions between lipid and CD molecules may lead to the quick rearrangement of the lipid membrane, accompanied by the swift release of CF molecules. The release of PDL from liposomes was maximized when the medication was encapsulated as a compound with β-CD. Nonetheless, the superior entrapment capacity of PDL as PDL/HP-β-CD complexes, compared to the unmodified drug, is an undeniable benefit of this method [44].

Surface-deacetylated chitin nanofibers (SDACNFs) reinforced with SBE-β-CD gel were formulated to serve as a controlled release carrier for PDL in the treatment of colitis. PDL was gradually liberated from the gel at both pH 1.2 and 6.8. The in vitro gradual release of PDL from the NFs-CDs gel was evidenced by the in vivo absorption of the medication following oral treatment to rats. The findings indicate that a basic gel formulated from a combination of SDACNFs and SBE-β-CD may be suitable for the regulated release of PDL. The injection of the NFs-CDs gel at 3-day intervals from the onset of DSS therapy led to a notable enhancement in both colitis symptoms and histological alterations in colon tissue. The therapeutic effects of the NFs-CDs gel on colitis were attributable to reduced neutrophil infiltration and diminished oxidative stress. The efficacy profiles of the NFs-CDs gel including PDL indicated its potential application in treating not just colitis but also various other conditions linked to inflammation and oxidative damage [80].

A two-photon aggregation-induced emission (AIE) active fluorophore (TP) has been synthesized and conjugated to β-CD through a ROS responsive bond, enabling the transport of prednisolone within its cavity via supramolecular interactions to form a diagnostic–therapeutic compound comprising two-photon PDL/fluorophore-CD complexes (TPCDP). Utilizing TPCDP encapsulated in nanosized micelles derived from a ROS-sensitive copolymer poly(2-methylthioethanol methacrylate)-poly(2-methacryloyloxyethyl phosphorylcholine), TPCDP@PMM can localize into atherosclerotic tissue via the compromised vascular endothelium. The micelles are disrupted by the locally overexpressed ROS and abundant lipids, leading to the further disintegration of TPCDP and the release of prednisolone. This occurs owing to the comparatively higher interaction between lipids and β-CD, which promotes anti-inflammatory effects and facilitates lipid clearance to reduce atherosclerosis. Additionally, designated as TP, TPCDP@PMM signifies a unique two-photon AIE image for the identification of atherosclerosis. The “two-pronged” therapeutic impact and plaque localization capability have been validated in vivo in ApoE^−/−^ mice, indicating that TPCDP@PMM holds significant promise for atherosclerosis theranostics [45].

Concluding, there is a large number of works on the complexes of PDL with CDs. Apart from the physicochemical and structural studies, indicating the stability of those inclusion complexes and 1:1 molar ratio in most cases, the number of articles on the pharmaceutical formulations including those systems is also quite large. It has been found that PDL when complexed with CDs can exhibit improved solubility and permeability, resulting in the increase bioavailability. This also affects the pharmacokinetic properties of this API, i.e., increases the AUC and T_max_. Those studies concern various routes of administration, not only oral but also topical and ocular.

### 5.4. Methylprednisolone

#### 5.4.1. Complexes with Different Cyclodextrins

Similar experiments to the ones conducted for PDL/CD complexes, described in Section 5.3.1, have been performed by the same group of authors, this time for 6a-methylprednisolone (Me-PDL) [40]. Phase-solubility diagrams of Me-PDL with various CDs at 25 °C were obtained. The intrinsic aqueous solubility of Me-PDL was determined to be 168 ± 5 mg/L. With the exception of γ-CD, all CDs exhibited A_L_-type diagrams within the designated concentration range. The γ-CD exhibited a B_S_-type diagram characterized by a steady and linear increase until it reached a concentration of 0.0125 M. The A_L_-type diagrams and the primary linear slope on the B_S_ diagrams exhibited a slope below one, suggesting the likely creation of 1:1 complexes in an aqueous solution [40].

The association constant between Me-PDL and γ-CD is more than twice as high as the association constant between Me-PDL and β-CD. This implies that the larger cavity of γ-CD is more advantageous for the formation of a compound with the bulkier Me-PDL compared to the cavity of β-CD. This provides additional evidence that the A-ring of these steroids is the primary location for binding with CDs [40].

The binding sites for Me-PDL were discovered at rings A (H1, H2, H4, and maybe H20), C (H11, H12), and D (H15, H16, H22, and possibly H14). However, the A-ring continues to be the primary binding site, albeit its binding shape has undergone alterations [40].

Unlike PDL, the complexes of Me-PDL showed contrasting behavior. The underivatized γ-CD formed more stable complexes compared to its SBE derivatives. The decreased size of the cavity opening could account for the lower association constants observed with Me-PDL in comparison to PDL. This is because the larger size of Me-PDL would make it more challenging for it to enter the cavity in contrast to the smaller size of PDL [40].

A separate group of researchers investigated the complexation in solution between methylprednisolone and three cyclodextrins: HP-β-CD, γ-CD, and HP-γ-CD. They employed phase solubility analysis, one and two-dimensional 1H-NMR, and molecular modeling techniques [41].

The solubility of Me-PDL significantly increased from 0.37 mM (0.13 mg/mL) to 27.9 mM (9.65 mg/mL) when the concentration of HP-β-CD was raised to 0.21 M (30%, *w*/*v*). Me-PDL’s solubility significantly increased from 0.37 mM (0.13 mg/mL) to 49.9 mM (17.28 mg/mL) when the concentration of HP-γ-CD was raised to 0.19 M (30%, *w*/*v*). The solubility of Me-PDL increases in the presence of HP-β-CD and HP-γ-CD in a linear manner. The resulting curve can be categorized as an A_L_-type (linear positive isotherm), which suggests the creation of water-soluble complexes. However, when the concentration of γ-CD was increased to 0.03 M (5%, *w*/*v*), there was an initial linear rise in the solubility of Me-PDL.

The subsequent addition of γ-CD led to a reduction in the solubility of Me-PDL. The solubility of Me-PDL in an aqueous solution of γ-CD follows a phase-solubility profile of the BS type. This suggests that the complexes formed between methylprednisolone and γ-CD have a restricted solubility in water [41].

The estimates of the complex formation constant (K_1:1_) indicate that the affinity of methylprednisolone to form complexes with CDs can be ranked as follows: γ-CD > HP-γ-CD > HP-β-CD [41].

Since ^1^H-NMR spectroscopy is a highly efficient technique for investigating the formation of inclusion complexes between CDs and various guest molecules, it has been widely employed to validate the structures of these complexes. The significant disparity in chemical shifts among protons situated within the hydrophobic cavity (H-30, H-50, and H-60), combined with the minimal variation in shifts among protons located on the outer region of γ-CD (H-10, H-20, and H-40), offers compelling proof of the formation of an inclusion complex [41].

The previously mentioned ^1^H-NMR observations suggest that there may be 1:1 complexes formed when the A and B rings of Me-PDL are included in the CD cavity, or when the C and D rings are included in the case of HP-β-CD. Alternatively, there could be a 1:2 complex formed between methylprednisolone and HP-β-CD, where the inclusion phenomena occur simultaneously in either direction of the Me-PDL molecule. Based on the outcome of the phase solubility analysis, which indicates that Me-PDL and HP-β-CD have an A_L_ profile, it is not reasonable to expect the development of a 1:2 complex. To accurately determine the stoichiometry of the complexes, the continuous variation method (Job’s plot) was utilized. Job’s plots consistently demonstrated a peak at r = 0.5, suggesting the presence of complexes with a 1:1 ratio within the concentration range under investigation. Based on the findings, the researchers suggested that there are 1:1 stoichiometry complexes formed between Me-PDL and three CDs. In these complexes, the A and B rings of methylprednisolone are inserted into the cavity of γ-CD and HP-γ-CD, whereas Me-PDL enters the cavity of HP-β-CD from either direction [41].

The molecular modeling study revealed that Me-PDL forms inclusion complexes with γ-CD and HP-γ-CD. This occurs when the A and B rings of methylprednisolone enter the CD cavity through its larger rim. The molecular modeling study was unable to be conducted for the HP-β-CD/Me-PDL complex. As a result, two potential scenarios for complex formation are suggested: (1) methylprednisolone enters HP-β-CD through its D and C rings from the wider rim, (2) the A and B rings of methylprednisolone penetrate deeper into the CD cavity, causing a portion of the A ring of the steroidal structure to remain outside of the cavity [41].

A different report presents the process of creating and analyzing novel cyclodextrin derivatives that contain both short alkyl ether (AE) chains (methyl (Me), ethyl (Et), or propyl (Pr)) and sulfoalkyl ether (SAE) chains on their main and secondary surfaces. This dual alteration should enable an increase in the height of the cavity, hence allowing these CDs to form complexes with various substrates while ideally preserving the safety characteristics of SAE cyclodextrin derivatives [81].

The efficacy of the novel CD derivatives was demonstrated by assessing their capacity to bind the Me-PDL. The analysis was conducted using both a UV/Vis technique and phase solubility analysis. The solubility of Me-PDL was enhanced when the amounts of SBE_7_-β-CD, SBE_5.2_-γ-CD, and SBE_5.2_-Et_4.9_-γ-CD were increased. Both Me-PDL substituents added to both SBE-β- and SBE-γ-CDs improved the binding of Me-PDL. This improvement was observed by both spectroscopic and phase solubility analysis. The binding constants increased as more ethyl substituents were added to both the β- and γ-series [81].

A similar study performed by the same researchers showed that regarding 6α-methylprednisolone, the substitution of propyl in SBE5.2-γ-CD resulted in a stronger binding compared to ethyl substitution, which in turn was stronger than methyl substitution. However, it is important to note that this trend was not observed with other steroids. This phenomenon is explained by the fact that in certain compounds, steric hindrance can impede both overall binding interactions and particular interactions. This hindrance can counteract the increased surface area caused by the alkyl chains on the CDs, thereby restricting the anticipated hydrophobic interactions [35].

#### 5.4.2. Therapeutic Application

Me-PDL is an intravenous or oral synthetic glucocorticoid medication. Me-PDL is frequently employed in the treatment of arthritis and various acute and chronic inflammatory conditions [82]. Administering methylprednisolone intravenously, however, results in a rapid clearance rate. An insufficient distribution of Me-PDL to the site of inflammation necessitates the administration of high and frequent doses (7.5 mg/day when taken orally daily, or up to 1000 mg every other day when administered intravenously) in order to obtain the desired therapeutic outcome [83]. An effective strategy to decrease the dosage, frequency of drug administration, and negative side effects, while still ensuring the drug’s effectiveness, is to create novel drug delivery systems that specifically target inflammatory sites and have a prolonged circulation duration [84].

**Figure 15:**
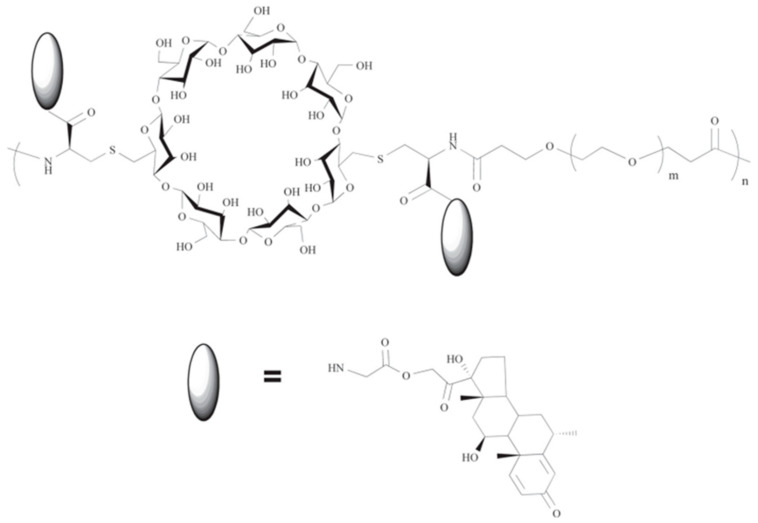
Schematic representation of the structure of CDP-MP, a conjugate of a glycinate derivative of α-methylprednisolone (MP) and a cyclodextrin polymer (CDP). m—number of ethylene glycol repeating units (average m = 77 for PEG with Mw 3400); n—number of repeating units of CDP-MP (average n = 24 ± 5 for parent polymer Mw of 117 kDa). Reprinted with permission from [85], licensed under CC BY 3.0.

The linear cyclodextrin polymer (CDP) consists of β-CD units linked by the polyethylene glycol. CDP easily dissolves in water, is compatible with living organisms, does not cause harm or toxicity, and does not trigger an immune response. CDP conjugates of hydrophobic small molecule medicines spontaneously form nanoparticles with sizes ranging from 30 to 50 nm (Figure 15) [85].

The scientists conducted a study on a nanoparticulate CDP conjugate of Me-PDL, where the medication is linked to the polymer through a glycinate ester. The efficacy of CDP-Me-PDL nanoparticles in inhibiting the growth of human lymphocytes was tested in vitro. Additionally, their effectiveness in a collagen-induced arthritis model was evaluated in vivo following intravenous administration [84].

A derivative of Me-PDL, namely, Me-PDL glycinate, was synthesized by esterifying glycine with the main alcohol group of Me-PDL. Subsequently, glycinate Me-PDL was chemically bonded to CDP. The conjugate was purified through the process of dialysis and then freeze-dried to obtain a white solid. The Me-PDL content bound to CDP was found to be 12.4% (*w*/*w*) with 0.08% of free Me-PDL, as measured by HPLC. The conjugation of Me-PDL with CDP resulted in a significant increase in the water solubility of Me-PDL, changing it from being insoluble (0.12 mg/mL) to highly soluble (more than 200 mg of CDP-MP/mL). The release kinetics of Me-PDL from the polymer conjugate exhibited a half-life (t_1/2_) of 50 h in a phosphate buffer solution (PBS) and 19 h in human plasma. When comparing CDP-Me-PDL with free Me-PDL, the proliferation of human lymphocytes was similarly reduced in vitro, although with a delayed impact. CDP-Me-PDL was intravenously delivered to animals with collagen-induced arthritis in vivo and compared to free Me-PDL. The complex CDP-Me-PDL was given once a week for a period of six weeks at doses of 0.07, 0.7, and 7 mg/kg/week. Additionally, it was given daily for the same duration at doses of 0.01, 0.1, and 1 mg/kg/day. There were little fluctuations in body weight among all animals. Animals that received a weekly treatment of an intermediate or high dose of CDP-Me-PDL showed a significant reduction in arthritis score after 28 days. In addition, animals treated with CDP-Me-PDL at the intermediate and high dose levels showed a reduction in dorsoplantar edema, returning it to its original level. The histological assessment revealed a decrease in synovitis, pannus development, and architectural disruption at the maximum dosage of CDP-Me-PDL. When provided regularly at similar cumulative doses, Me-PDL demonstrated modest efficacy in this model [84]. This study illustrates that the coupling of Me-PDL to a cyclodextrin polymer can enhance its effectiveness, resulting in the need for lower doses and less frequent administration. This offers a safer and more convenient approach to managing rheumatoid arthritis [84].

## 6. Conclusions

Due to their low polarity and appropriate molecular size, hydrocortisone-type corticosteroids can form stable (K > 10^3^) inclusion complexes with multiple cyclodextrins by interacting with their hydrophobic cavities. This review demonstrates that such complexes have been extensively synthesized and analyzed for over 50 years, with the number of such works constantly increasing. Apart from structural and physicochemical studies, focused on the determination of the complex ratio, stability, and CD–HTC interactions, a lot of application-related works have been published as well. Precisely, CDs have been successfully used as solubilizers and absorption enhancers in drug formulations, including various routes of administration such as oral, ocular, and topical.

Undoubtedly, hydrocortisone, prednisolone, and methylprednisolone are the three most widely studied HTCs in terms of their complexes with CDs. This is caused by the beneficial pharmacodynamic properties of those APIs resulting in their widespread use.

However, despite the extensive characterization of numerous CD–HTC complexes, the diversity of both native and substituted CDs suggests that there remains significant potential for further exploration in this area. This is mostly attributable to the fact that the specific type of CD employed for complexation significantly affects the characteristics of the resultant complex, including the enhancement of the guest molecule’s dissolution, the host–guest ratio, and the stability of the complex. For example, recent studies show that the stoichiometry of the host–guest complex for a particular CD/HTC pair is not always constant, with its value being influenced by multiple factors such as the temperature, choice of solvent, concentration of reactants, or presence of other molecules in the solution. Furthermore, to the best of our knowledge, some of the HTCs, such as, i.e., tixocortol pivalate, have not been studied in terms of their ability to form inclusion complexes with CDs, while the others, i.e., prednisone, have been analyzed using only theoretical methods. Consequently, we anticipate that this study will assist in planning such studies and enable the comparison of freshly acquired results with previously published findings.

## Figures and Tables

**Table 1:** Complexes of hydrocortisone-type corticosteroids and cyclodextrins and the methods used to analyze them. The host–guest ratio column provides information on the ratio values mentioned in the following papers. The value should not be interpreted as the ratio used in the method of analysis.

Corticosteroid (Guest)	Cyclodextrin (Host)	Method of Analysis	Host–Guest Ratio of the Complex	Source
cortisol	β-CD	NMR	2:1	[13]
cortisone	β-CD	phase solubility diagrams; MS; chemical analysis of pure the pure solid complexes	1:1; 2:1	[14]
cortisone acetate	RM-β-CD-CA	phase solubility diagrams, NMR, DSC	1:1	[15]
cortisone acetate	SBE-β-CD-CA	phase solubility diagrams, NMR, DSC	1:1	[15]
cortisone acetate	HP-β-CD	phase solubility diagrams, NMR, DSC	1:1	[15]
cortisone acetate	β-CD	phase solubility diagrams, NMR, DSC	2:1	[15]
cortisone acetate	HP-β-CD	MAS spectroscopy	1:1; 2:1	[16]
hydrocortisone	β-CD	phase solubility diagrams; MS; chemical analysis of pure the pure solid complexes	1:1; 2:1	[14]
hydrocortisone	HP-β-CD	phase solubility diagrams	1:1	[17]
hydrocortisone	γ-CD	phase solubility diagram, HPLC	1:1	[18]
hydrocortisone	HP-β-CD	phase solubility diagrams	1:1	[19]
hydrocortisone	HP-β-CD	SEM, FTIR, 1H NMR, phase solubility diagrams	1:1; 1.5:1; 2:1	[20]
hydrocortisone	HP-β-CD	DSC, HPLC	2:1	[21]
hydrocortisone	β-CD	DSC, HPLC	2:1	[21]
hydrocortisone	β-CDC6	DSC, FTIR	1:1	[22]
hydrocortisone	HP-β-CD	HPLC	2:1	[23]
hydrocortisone	M4βCD	phase solubility diagrams	1:1	[24]
hydrocortisone	βCD	phase solubility diagrams	1:1	[24]
hydrocortisone	β-CD		1:1; 2:1	[25]
hydrocortisone	HP-β-CD		1:1	[26]
hydrocortisone	γ-CD	phase solubility diagrams, ITC	1:1; 3:2	[27]
hydrocortisone	HP-β-CD	HPLC, total viable aerobic count	2:1; 4:1; 6:1; 7:1; 9:1; 10:1	[14]
hydrocortisone	γ-CD	HPLC	1:1	[28]
hydrocortisone	HP-β-CD		1:1	[29]
hydrocortisone	β-CD	solubility studies, HPLC	2:1	[30]
hydrocortisone	γ-CD	HPLC, phase solubility diagrams	1:1	[31]
hydrocortisone	80/20 gCD/HPgCD	HPLC, phase solubility diagrams	1:1	[31]
hydrocortisone	β-CD		1:1	[32]
hydrocortisone	α-CD	phase solubility diagrams, HPLC	1:1	[33]
hydrocortisone	β-CD	phase solubility diagrams, HPLC	1:1	[33]
hydrocortisone	γ-CD	phase solubility diagrams, HPLC	1:1	[33]
hydrocortisone	HP-β-CD	phase solubility diagrams, HPLC	1:1	[33]
hydrocortisone	HP-γ-CD	phase solubility diagrams	1:1	[34]
hydrocortisone	γ-CD	phase solubility diagrams	1:1	[34]
hydrocortisone	SBE6.5-b-CD	UV spectrophotometry	1:1	[35]
hydrocortisone	γ-CD	UV spectrophotometry	1:1	[35]
hydrocortisone	Et4,5γ-CD	UV spectrophotometry	1:1	[35]
hydrocortisone	SBE 5,2 γ-CD	UV spectrophotometry	1:1	[35]
hydrocortisone	SBE 5,2 Et 4,9 γ-CD	UV spectrophotometry	1:1	[35]
hydrocortisone	SBE 5,2 Et 6,9 γ-CD	UV spectrophotometry	1:1	[35]
hydrocortisone	SBE 5,2 Et 8,9 γ-CD	UV spectrophotometry	1:1	[35]
hydrocortisone	SBE 4,6β-CD	UV spectrophotometry	1:1	[35]
hydrocortisone	SBE 4,6 Et 3,5 β-CD	UV spectrophotometry	1:1	[35]
hydrocortisone	SBE 4,6 Et 6,5 β-CD	UV spectrophotometry	1:1	[35]
hydrocortisone	SBE 4,6 Et 8,5 β-CD	UV spectrophotometry	1:1	[35]
hydrocortisone	HP-β-CD	UV spectrophotometry	1:1	[35]
hydrocortisone	SBE 5,2 Pr 5,4 γ-CD	UV spectrophotometry	1:1	[35]
hydrocortisone	SBE 5,2 Me 5,2 γ-CD	UV spectrophotometry	1:1	[35]
hydrocortisone	HP-β-CD	phase solubility diagrams	1:1	[19]
hydrocortisone	β-CD		1:1; 2:1; 4:1; 0.5:1	[36]
hydrocortisone	HP-β-CD	HPLC	1:1	[37]
hydrocortisone	HP-β-CD	HPLC	4:1	[38]
hydrocortisone	(cat CD	HPLC	4:1	[38]
hydrocortisone	HP-β-CD		3:1	[39]
methylprednisolone	β-CD	NMR; phase solubility diagrams	1:1	[40]
methylprednisolone	γ-CD	1H NMR; phase solubility diagrams; Job’s plots	1:1	[41]
methylprednisolone	HP-γ-CD	1H NMR; phase solubility diagrams; Job’s plots	1:1	[41]
methylprednisolone	HP-β-CD	1H NMR; phase solubility diagrams; Job’s plots	1:1	[41]
methylprednisolone	SBE6.5-b-CD	UV spectrophotometry	1:1	[35]
methylprednisolone	γ-CD	UV spectrophotometry	1:1	[35]
methylprednisolone	Et4,5γ-CD	UV spectrophotometry	1:1	[35]
methylprednisolone	SBE 5,2 γ-CD	UV spectrophotometry	1:1	[35]
methylprednisolone	SBE 5,2 Et 4,9 γ-CD	UV spectrophotometry	1:1	[35]
methylprednisolone	SBE 5,2 Et 6,9 γ-CD	UV spectrophotometry	1:1	[35]
methylprednisolone	SBE 5,2 Et 8,9 γ-CD	UV spectrophotometry	1:1	[35]
methylprednisolone	SBE 4,6β-CD	UV spectrophotometry	1:1	[35]
methylprednisolone	SBE 4,6 Et 3,5 β-CD	UV spectrophotometry	1:1	[35]
methylprednisolone	SBE 4,6 Et 6,5 β-CD	UV spectrophotometry	1:1	[35]
methylprednisolone	SBE 4,6 Et 8,5 β-CD	UV spectrophotometry	1:1	[35]
methylprednisolone	HP-β-CD	UV spectrophotometry	1:1	[35]
methylprednisolone	SBE 5,2 Pr 5,4 γ-CD	UV spectrophotometry	1:1	[35]
methylprednisolone	SBE 5,2 Me 5,2 γ-CD	UV spectrophotometry	1:1	[35]
prednisolone	β-CD	NMR; phase solubility diagrams	1:1	[40]
prednisolone	β-CD	dissolution studies, membrane permeation studies, in vivo absorption studies	2:1	[42]
prednisolone	(SBE)7M-b-CD	UV spectrophotometry	1:1	[43]
prednisolone	HP-β-CD		1:1	[26]
prednisolone	β-CD	DSC, X-ray diffractometry, and FT-IR spectroscopy	8.4:1	[44]
prednisolone	HP-β-CD	DSC, X-ray diffractometry, and FT-IR spectroscopy	2:1; 6:1	[44]
prednisolone	two-photon fluorophoreβ-cyclodextrin	2D-NOESY NMR	1:1	[45]
prednisolone	SBE6.5-b-CD	UV spectrophotometry	1:1	[35]
prednisolone	γ-CD	UV spectrophotometry	1:1	[35]
prednisolone	Et4,5γ-CD	UV spectrophotometry	1:1	[35]
prednisolone	SBE 5,2 γ-CD	UV spectrophotometry	1:1	[35]
prednisolone	SBE 5,2 Et 4,9 γ-CD	UV spectrophotometry	1:1	[35]
prednisolone	SBE 5,2 Et 6,9 γ-CD	UV spectrophotometry	1:1	[35]
prednisolone	SBE 5,2 Et 8,9 γ-CD	UV spectrophotometry	1:1	[35]
prednisolone	SBE 4,6β-CD	UV spectrophotometry	1:1	[35]
prednisolone	SBE 4,6 Et 3,5 β-CD	UV spectrophotometry	1:1	[35]
prednisolone	SBE 4,6 Et 6,5 β-CD	UV spectrophotometry	1:1	[35]
prednisolone	SBE 4,6 Et 8,5 β-CD	UV spectrophotometry	1:1	[35]
prednisolone	HP-β-CD	UV spectrophotometry	1:1	[35]
prednisolone	SBE 5,2 Pr 5,4 γ-CD	UV spectrophotometry	1:1	[35]
prednisolone	SBE 5,2 Me 5,2 γ-CD	UV spectrophotometry	1:1	[35]
prednisolone	α-CD	NMR, HPLC	1:1	[46]
prednisolone	β-CD	NMR, HPLC	1:1	[46]
prednisolone	γ-CD	NMR, HPLC	1:1	[46]
prednisolone	β-CD	HPLC	2:1	[47]
prednisolone	γ-CD	HPLC	3:2	[47]
prednisolone	β-CD	dissolution studies, membrane permeation studies, in vivo absorption studies	2:1	[42]
prednisolone	(SBE)7m-βCD	HPLC	1:1; 2:1	[48]
prednisolone	HP-β-CD	HPLC	2:1	[48]
prednisolone	(SBE)7m-β-CD	X-ray, release profiles	1:1; 2:1; 3:1	[43]
prednisolone	HP-β-CD	phase solubility diagrams	1:1	[49]
prednisolone	HP-γ-CD	phase solubility diagrams	1:1	[49]
prednisone	γ-CD	in silico interaction energy study	1:1	[50]

**Table 2:** Apparent stability constant (K_1:1_) and complexation efficiency (CE) values of hydrocortisone/CD complexes.

Cyclodextrin	Slope	Correlation Coefficient	K_1:1_	CE
α-CD	0.062	0.993	72	0.07
βCD	0.542	0.999	1360	1.18
γCD	0.632	0.999	1970	1.72
HP-β-CD	0.558	0.998	1450	1.26
SBE-β-CD	0.580	0.999	1580	1.38
HP-γ-CD	0.516	0.998	1270	1.07

**Table 3:** The apparent complexation constant and CE of various drugs in eye-drop preparations.

Cyclodextrin	γCD/HPγCD Ratio	CE (Individual)	CE (Combination)			
			Dexamethasone		Second Drug	
			CE Value	CE Ratio	CE Value	CE Ratio
Dexamethasone						
γCD	-	0.26	-	-	-	-
γCD/HPγCD	80/20	0.69	-	-	-	-
γCD/HPγCD	20/80	1.26	-	-	-	-
HPγCD	-	1.07	-	-	-	-
Hydrocortisone						
γCD	-	0.31	0.07	0.28	0.11	0.35
γCD/HPγCD	80/20	1.90	0.24	0.35	0.32	0.17
γCD/HPγCD	20/80	1.61	0.48	0.38	0.74	0.46
HPγCD	-	1.21	0.45	0.42	0.68	0.56

**Table 4:** The solution properties and the fiber diameters of the resulting electrospun nanofibers.

Sample	HP-β-CD Concentration (%, *w*/*v*)	Molar Ratio of HC/HP-β-CD	Viscosity (Pa S)	Conductivity (μS cm^−1^)	Average Fiber Diameter (nm)
HP-β-CD	200	-	1.533	36.3	215 ± 65
HC/HP-β-CD (1:1)	180	1:1	1.395	37.6	190 ± 55
HC/HP-β-CD (1:1.5)	180	1:1.5	1.339	34.7	330 ± 165
HC/HP-β-CD (1:2)	180	1:2	1.156	28.9	375 ± 160

**Table 5:** The influence of γ-CD on permeation flux (J, μg/mL∙h∙cm^2^) of three model drugs, defined by the flux enhancement ratio (J_R_).

Membrane	γ-CD, % (*w*/*v*)	Hydrocortisone	
		Flux (J)	Ratio (J_R_)
Cellophane	0 10	12.7 ± 1.19 230 ± 5.64	18.1
Cellophane with mucin	0 10	12.6 ± 1.06 234 ± 9.30	18.6
Dual	0 10	3.65 ± 0.24 70.9 ± 15.2	19.4
Dual with mucin	0 10	4.41 ± 0.25 82.9 ± 22.7	18.8

## Abbreviations

API	active pharmaceutical ingredient
BK	benzalkonium chloride
CA	cortisone acetate
CAD	charged aerosol detector
CAT-CD	2-hydroxy-3-trimethyl-ammoniopropyl-cyclodextrin
CD	cyclodextrin
CDP	linear cyclodextrin polymer
CE	complexation efficiency
CF	5,6-carboxyfluorescein
CM-β-CD	carboxymethyl-beta-cyclodextrin
CO	cortisone
DAD	diode array detector
DLS	dynamic light scattering
DSC	differential scanning calorimetry
FTIR	Fourier transform infrared
HA	hyaluronic acid
HC	hydrocortisone
HCA	hydrocortisone acetate
HP	hydroxypropyl
HPLC	high-pressure liquid chromatographic
HTC	hydrocortisone-type corticosteroid
Me-PDL	methylprednisolone
ML-β-CD	maltosyl-beta-cyclodextrin
MSPM	magnetic solid-phase microextraction
NMR	nuclear magnetic resonance
PA	prednisone acetate
PC	phosphatidylcholine
PDL	prednisolone
PG	1,2-propylene-glycol
PNA	prednisolone acetate
PPG	polypropylene glycol
PVP	polyvinylpyrrolidone
R_M_	resistance of the membrane
RM-β-CD	randomly methylated beta cyclodextrin
SBE	sulfobutyl ether
SBE-AE-CD	sulfobutyl ether–alkyl ether
SEM-EDS	scanning electron microscopy–electron diffraction spectroscopy
SLN	solid lipid nanoparticles
TEM	transmission electron microscopy
TPCDP	two-photon fluorophore-CD/PDL
UWL	unstirred water layer
XRD	X-ray diffraction
ZnGlu	zinc gluconate
ZnHA	zinc hyaluronate
β-CDC6	amphiphilic β-CD
